# VEGFA/NRP-1/GAPVD1 axis promotes progression and cancer stemness of triple-negative breast cancer by enhancing tumor cell-macrophage crosstalk

**DOI:** 10.7150/ijbs.86085

**Published:** 2024-01-01

**Authors:** Lu Wang, Lifen Zhang, Lin Zhao, Shan Shao, Qian Ning, Xin Jing, Yujiao Zhang, Fengyu Zhao, Xizhi Liu, Shanzhi Gu, Xinhan Zhao, Minna Luo

**Affiliations:** 1 Department of Oncology, The First Affiliated Hospital of Xi'an Jiaotong University, Xi'an, Shaanxi Province, 710061, China.; 2 Department of Respiratory, The First Affiliated Hospital of Xi'an Jiaotong University, Xi'an, Shaanxi Province, 710061, China.; 3 Department of Pathology, Shaanxi Provincial People's Hospital, Xi'an, Shaanxi Province, 710068, China.; 4 Department of Respiratory and Critical Care Medicine, The Second Affiliated Hospital of Xi'an Jiaotong University, Xi'an, Shaanxi Province, 710004, China.; 5 Department of Forensic Medicine, Medical School of Xi'an Jiaotong University, Xi'an, Shaanxi Province, 710061, China.; 6 Department of Hematology, The First Affiliated Hospital of Xi'an Jiaotong University, Xi'an, Shaanxi Province, 710061, China.

**Keywords:** Triple-negative breast cancer, cancer stem cell, VEGFA/NRP-1/GAPVD1 axis, tumor-associated macrophage.

## Abstract

Triple-negative breast cancer (TNBC) has long been considered a major clinical challenge due to its aggressive behavior and poor prognosis. Cancer stem cells (CSCs) are known as the main cells responsible for tumor origination, progression, recurrence and metastasis. Here, we report that M2-type tumor-associated macrophages (TAMs) contribute to cancer stemness in TNBC cells via the secretion of VEGFA. Reciprocally, elevated VEGFA expression by TAM-educated TNBC cells acts as a regulator of macrophage polarization, therefore constitute a feed-back loop between TNBC cells and TAMs. Mechanistically, VEGFA facilitates the CSC phenotype via the NRP-1 receptor and downstream GAPVD1/Wnt/β-catenin signaling pathway in TNBC cells. Our study underscores the crosstalk between TNBC cells and TAMs mediated by VEGFA and further clarifies the role and underlying mechanisms of the VEGFA/NRP-1/GAPVD1 axis in regulating cancer stemness. We also document an immunosuppressive function of VEGFA in the tumor microenvironment (TME). Therefore, the present study indicates crosstalk between TNBC cells and TAMs induced by VEGFA and provides a potential implication for the combination of immunotherapy and VEGFA-targeted agents in TNBC therapy.

## Introduction

Breast cancer is the most common female malignancy worldwide 1. Triple-negative breast cancer (TNBC) is the most aggressive subtype of breast cancer, accounting for 15-20% of breast cancers 2. Due to the poor prognosis and lack of effective targeted therapy, it is extremely urgent to find novel and effective targets for TNBC treatment.

Macrophages have emerged as a major component of the tumor microenvironment (TME) and are closely related to the development of tumors 3. In the breast cancer TME, tumor-associated macrophages (TAMs) are one of the most abundant immune cells 4. Breast tumors and their immunoenvironment are interwoven entities, and both are important for tumor progression 5, 6. In breast cancer, high TAM density indicates poor prognosis 7. In TNBC, the presence of TAMs and TNBC cells with high CD68^+^ TAMs/low CD8^+^ tumor-infiltrating lymphocytes (TILs) was correlated with significantly shorter RFS and OS and indicated poorer prognosis 8. Li H *et al.* 9 reported that TAM-derived TGF-β1 induced ferroptosis resistance in TNBC cells, reciprocally, elevated IL-6 expression by TAM-educated TNBC cells significantly promoted the recruitment of macrophages in a feedback mechanism. These results indicated tight crosstalk between tumor cells and TAMs in TNBC. However, the mechanisms underlying the crosstalk between tumor cells and TAMs in TNBC remain unclear.

TAMs are an important source of vascular endothelial growth factor A (VEGFA). VEGFA is the main driver of angiogenesis in the tumor microenvironment in several cancers. In addition to its role in angiogenesis, VEGFA also contributes to key aspects of tumorigenesis in several kinds of malignancies, including renal cell carcinoma, lung cancer, and colorectal cancer 10. Cao *et al.* 11 defined VEGFA exerted an angiogenesis-independent function in renal cell carcinoma cells to promote their malignant progression through the neuropilin-1 (NRP-1) receptor. Our previous study found that the VEGF/NRP-1 axis may be involved in the regulation of migration, invasion, and EMT transformation in breast cancer 12. These findings highlighted the importance of VEGFA in tumor cells in the context of their role in promoting the progression of tumors.

NRP-1 is a transmembrane glycoprotein receptor that interacts with class 3 semaphorins and members of the VEGF ligand family. NRP-1 was found to be elevated in multiple malignant tumors, such as breast cancer, lung cancer and gastric cancer 13-15, and participated in the promotion of tumor proliferation, epithelial-mesenchymal transition (EMT), invasion and migration 16-18. A common feature of TNBC is its EMT phenotype. EMT generates cells with properties of stem cells 19, 20. CSCs are considered the main cells responsible for tumor origination, progression, recurrence and metastasis 21. Previous studies demonstrated that VEGF-A acted via NRP-1 to enhance epidermal cancer stem cell survival and promoted the formation of aggressive and highly vascularized tumors 22. Angom RS *et al.* 23 observed that patient-derived GBM cells expressing shRNA against VEGF or NRP-1 attenuated cancer stem cell markers and inhibited tumor-initiating cell neurosphere-forming capacity and migration. Tang *et al.* 24 reported that NRP-1 was overexpressed in claudin-low breast cancer and promoted tumor progression through the acquisition of stem cell characteristics and RAS/MAPK pathway activation. These studies implicated VEGFA and its receptor NRP-1 contributed to the self-renewal and survival of CSCs. Nevertheless, the roles and detailed mechanisms of the VEGFA/NRP-1 axis in TNBC stemness have not been clearly defined.

The current literature supports an important function for VEGFA in TNBC progression. TAMs represent one of the most predominant sources of VEGFA in the TME. However, the precise role of TAM-derived VEGFA and its downstream mechanisms underlying the crosstalk between tumor cells and TAMs are still largely unknown. Therefore, we set out to investigate the contribution of VEGFA to TNBC CSC functions and its role in tumor cell-macrophage crosstalk.

## Results

### TAMs promote the migration, invasion and cancer stemness of TNBC cells *in vitro*

**Figure 1:**
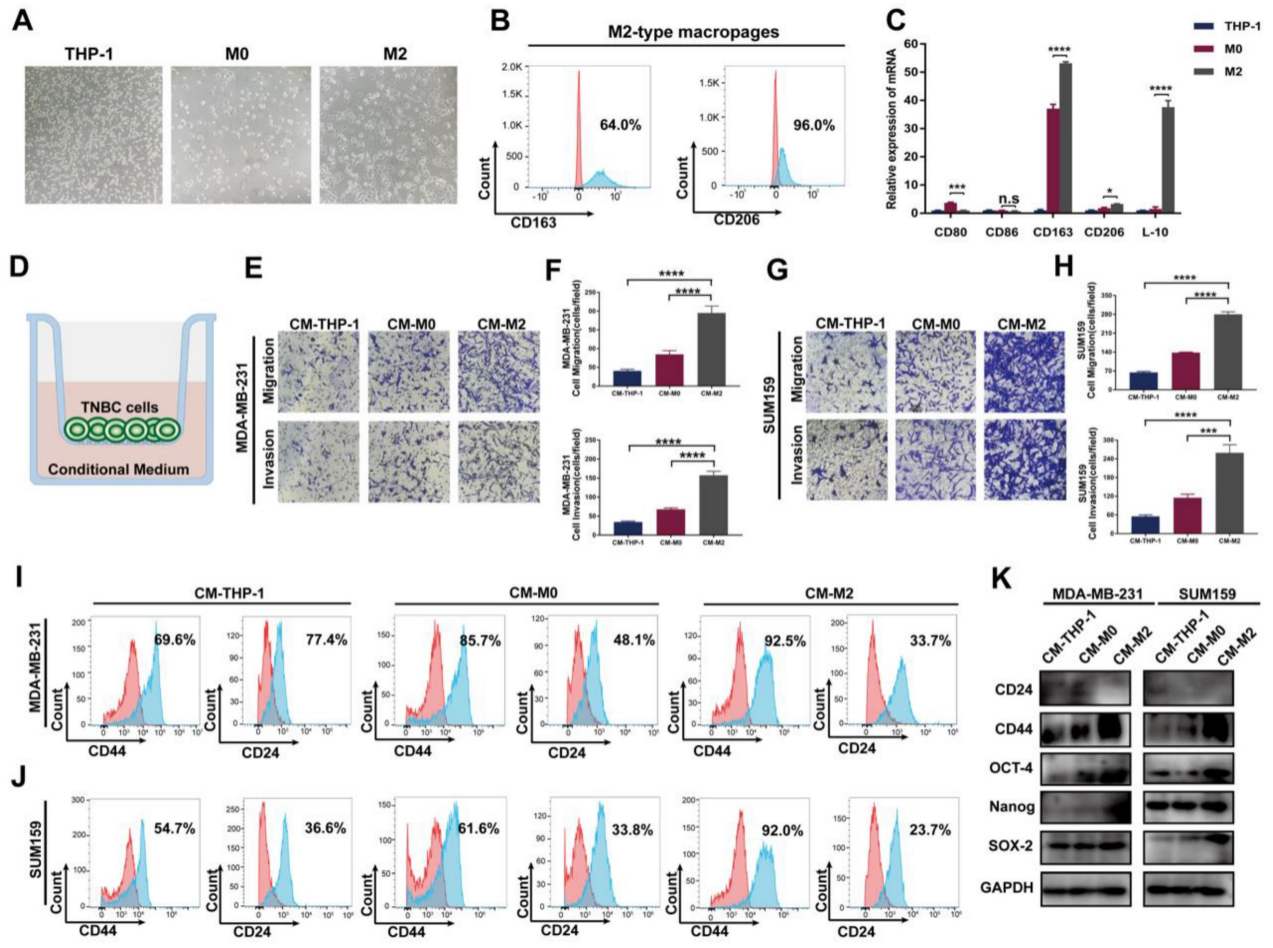
** TAMs promote migration, invasion and cancer stemness of TNBC *in vitro.* (A)** The cell morphology of THP-1, M0 and M2 type macrophages was observed by microscopy (100 ×). **(B)** Flow cytometry analysis of the expression of the M2 macrophage markers CD163 and CD206. **(C)** The mRNA expression of M1-related markers (CD80 and CD86) and M2-related markers (CD163, CD206 and IL-10) was evaluated by RT‒qPCR in THP-1 cells, M0-type macrophages and M2-type macrophages. **(D)** Schematic chart showing the coculture system. (E-H) Migration and invasion of TNBC cells after coculture with the indicated conditional medium were observed using Transwell assay (200 ×). The error bar indicates the mean ± SD. **(I-J)** Flow cytometry analysis of CD44 and CD24 expression on TNBC cells after coculture with the indicated conditional medium from THP-1 cells, M0-type macrophages and M2-type macrophages. **(K)** Western blotting of breast cancer stem cell markers (CD24, CD44, OCT-4, Nanog and SOX-2) after coculture with the indicated conditioned medium. The images show representative data, and data are expressed as the mean ± SD of each group of cells from three separate experiments. n.s., no significance, *P < 0.05, ***P < 0.001, ****P < 0.0001 vs. the controls.

To evaluate the impact of TAMs on TNBC cells *in vitro,* we first successfully induced M2-type macrophages according to the established method 25 (Figure 1A-C). Then, the conditional medium of THP-1 monocytes, M0 and M2 macrophages was collected and cocultured with TNBC cells. As shown in Figure 1D-H, compared to THP-1 cell group, the migration and invasion abilities were significantly increased after incubation with M2-type macrophage-derived conditional medium, indicating an enhancement of cancer aggressiveness after coculture with TAMs. Previous literature indicates that TAMs contribute to tumor progression by promoting genetic instability, supporting metastasis and nurturing cancer stem cells 26. Therefore, we next detected the CSC phenotype in TNBC cells. As shown in Figure 1I-J, FACS showed a significant increase in the fraction of the CD44^+^/CD24^-^ cell subpopulation in the two TNBC cell lines. Consistent with these data, western blotting showed that the expression of the CSC markers CD44, OCT-4, Nanog and SOX-2 markedly increased in the group treated with M2 macrophage-derived conditional medium, whereas CD24 levels notably decreased (Figure 1K). These findings indicate that TAMs promote the migration, invasion and cancer stemness of TNBC cells *in vitro.*

### VEGFA is selectively highly expressed in TAMs and TNBC cells and generates TNBC cells with a CSC phenotype via NRP-1

**Figure 2:**
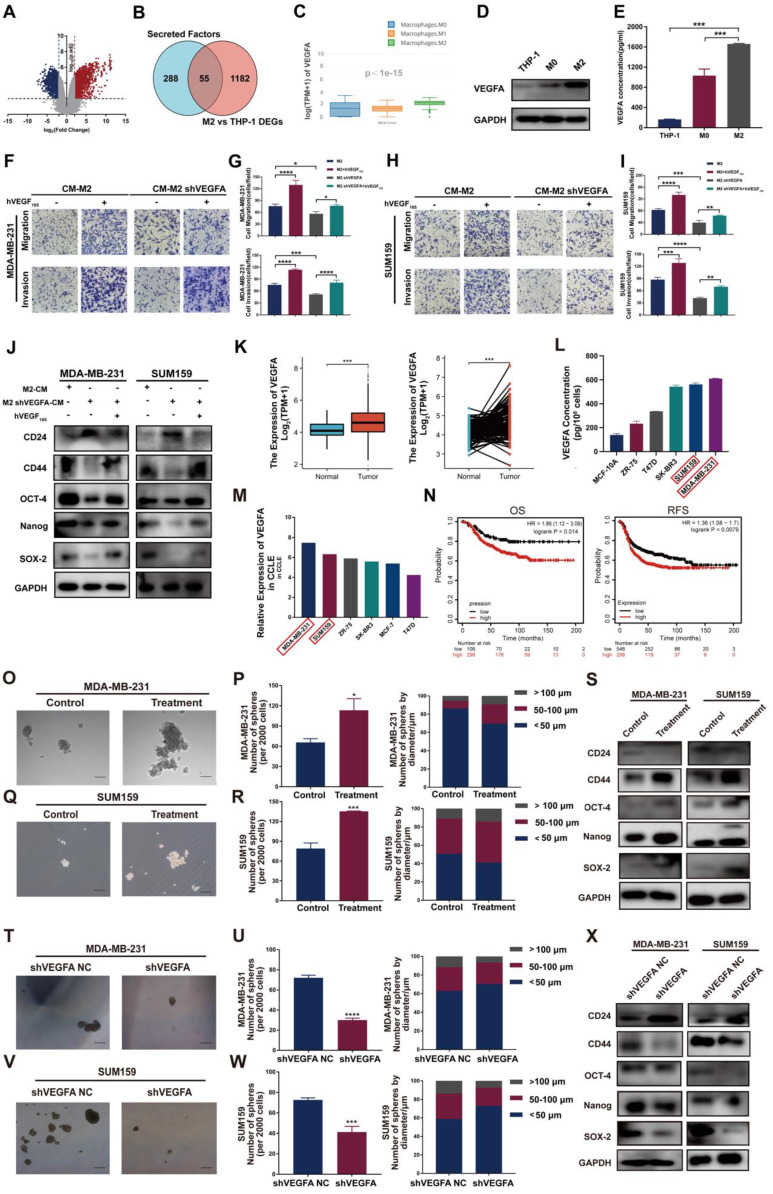
** VEGFA is highly expressed in M2 type TAMs and TNBC cells and generates TNBC cells with CSC phenotype. (A)** Volcano plot representing the differentially expressed genes between THP-1 and M2-type macrophages. **(B)** Venn diagram representing the differentially expressed genes overlapping between the GEO database and GeneCard database. Blue: secretory factor gene sets in the GeneCard database. Red: differentially expressed genes between THP-1 monocytes and M2-like macrophages in the GEO database. **(C)** VEGFA expression in M0-, M1- and M2-type macrophages of breast cancer using the GEPIA2021 database. **(D)** Western blotting analysis of VEGFA expression in THP-1 cells and M0- and M2-type macrophages. **(E)** ELISA detection of the secretion of VEGFA in THP-1 cells and M0- and M2-type macrophages. (F-I) Migration and invasion of TNBC cells after coculture with the indicated conditional medium were determined by Transwell assay (200 ×). The error bar indicates the mean ± SD. **(J)** Western blotting of breast cancer stem cell markers (CD24, CD44, OCT-4, Nanog and SOX-2) in TNBC cells after coculture with the indicated conditioned medium in the presence or absence of 10 ng/ml hVEGF_165_. **(K)** VEGFA mRNA expression in nonpaired (left panel, adjacent noncancerous tissue n=113, cancer tissue n=1113) and paired (right panel, n = 113) breast cancer samples from the TCGA database. The error bar indicates the mean ± SD. **(L)** The secretion of VEGFA from different breast cancer cell lines was assessed by ELISA. **(M)** VEGFA expression in different breast cancer cell lines from the CCLE database. **(N)** Kaplan-Meier analysis to compare the OS (high n=298, low n=106) and RFS (high n=298, low n=548) of TNBC patients with high and low VEGFA mRNA expression using the TCGA database.** (O-R)** Representative images of the microspheres formed after treatment of TNBC cells with 10 ng/ml hVEGF_165_ (Treatment). The number of microspheres was counted and plotted, and the percentage of microspheres with diameters of < 50 μm, 50-100 μm and > 100 μm was calculated and plotted (200 ×, scale bars = 100 μm). **(S)** Western blotting of breast cancer stem cell markers (CD24, CD44, OCT-4, Nanog and SOX-2) in TNBC cells after treatment with 10 ng/ml hVEGF_165_.** (T-W)** Representative images of the microspheres after VEGFA knockdown (shVEGFA) in TNBC cells. The number of microspheres was counted and plotted, and the percentage of microspheres with diameters of < 50 μm, 50-100 μm and > 100 μm was calculated and plotted (200 ×, scale bars = 100 μm). **(X)** Western blotting of breast cancer stem cell markers (CD24, CD44, OCT-4, Nanog and SOX-2) in TNBC cells after VEGFA knockdown. The images show representative data, and data are expressed as the mean ± SD of each group of cells from three separate experiments. n.s., no significance, *P < 0.05, **P < 0.01, ***P < 0.001, ****P < 0.0001 vs. the controls.

To explore the molecular mechanism by which TAMs promote cancer invasiveness and stemness in TNBC cells, we first identified sets of genes differentially expressed between THP-1 monocytes and M2-type macrophages using the GEO dataset. The analysis of GSE52292 revealed 1237 differentially expressed genes between M2-type macrophages and THP-1 monocytes (Table S1 and Figure 2A). Then, GeneCard was analyzed to retrieve a collection of secretory factor gene sets (Table S2), and the overlapping genes of the two sets above were analyzed (Table S3 and Figure 2B). The results showed 55 differentially expressed genes, including VEGFA. GEPIA2021 website analysis further showed that the expression of VEGFA was notably heightened in M2-type macrophages compared to other types of macrophages (Figure 2C). Western blotting and ELISA confirmed that the VEGFA expression levels in M2-type macrophages and their supernatant exhibited a remarkable increase compared to THP-1 monocytes and M0 macrophages (Figure 2D-E). These results indicate that VEGFA is selectively highly expressed in M2-type macrophages. Thus, we speculate that VEGFA secreted by M2-type macrophages may be positively associated with breast cancer mobility and stemness.

Next, we validated the impact of TAM-derived VEGFA on TNBC cell invasiveness and stemness. Exogenous hVEGF_165_ increased the numbers of migrated and invaded cells and markedly increased the levels of the CSC markers CD44, OCT-4, Nanog and SOX-2. When M2-type macrophages with VEGFA knockdown were cocultured with TNBC cells, a remarkable decrease in the CSC markers CD44, OCT-4, Nanog and SOX-2 and an apparent increase in CD24 expression in TNBC cells were observed. When we reintroduced exogenous hVEGF_165_ into the coculture system, CSC marker expression was restored (Figure 2F-J, S1A-B). Taken together, these results indicate that TAM-derived VEGFA contributes to CSC sustenance in TNBC cells.

We next detected VEGFA expression and its impact on TNBC cells. Similarly, in TNBC tissue and cell lines, the expression of VEGFA was significantly higher than that in other molecular subtypes and predicted poor prognosis (Figure 2K-N). Next, the effect of VEGFA on TNBC cell stemness was examined through several assays.

Sphere formation assay demonstrated that 10 ng/ml exogenous hVEGF_165_ successfully promoted microsphere formation *in vitro* (Figure 2O-R). The expression of the CSC markers CD44, OCT-4, Nanog and SOX-2 markedly increased in the group treated with exogenous hVEGF_165_, whereas CD24 levels notably decreased (Figure 2S). In contrast, knockdown of VEGFA expression significantly attenuated microsphere formation and inhibited the expression of CSC markers (Figure 2T-X, S1C-F). Transwell and wound healing assays showed that VEGFA strongly promoted the migration and invasion of TNBC cells (Figure S2A-H), while knockdown of VEGFA greatly attenuated migration and invasion (Figure S2I-P).

**Figure 3:**
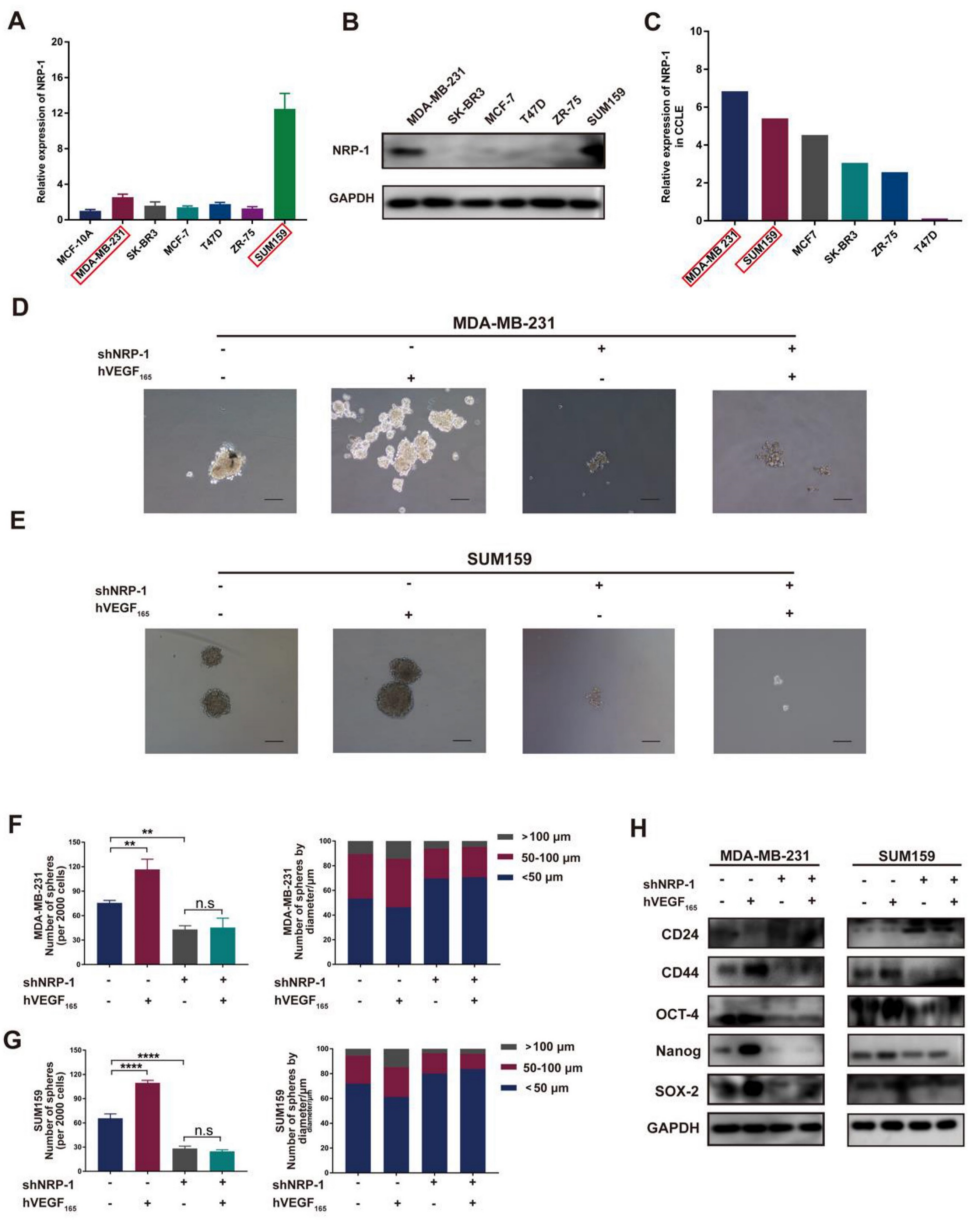
** VEGFA promotes the CSC phenotype via NRP-1. (A-B)** The mRNA and protein levels of NRP-1 in different breast cancer cell lines were detected by RT‒qPCR and Western blotting. **(C)** NRP-1 mRNA expression in different breast cancer cell lines from the CCLE database. **(D-G)** Representative images of microspheres after NRP-1 knockdown (shNRP-1) in the presence or absence of 10 ng/ml hVEGF_165_. The number of microspheres was counted and plotted, and the percentage of microspheres with diameters of < 50 μm, 50-100 μm and > 100 μm was calculated and plotted (200 ×, scale bars = 100 μm). **(H)** Western blotting of breast cancer stem cell markers (CD24, CD44, OCT-4, Nanog and SOX-2) in TNBC cells after NRP-1 knockdown in the presence or absence of 10 ng/ml hVEGF_165_. The images show representative data, and data are expressed as the mean ± SD of each group of cells from three separate experiments. n.s., no significance, **P < 0.01, ****P < 0.0001 vs. the controls.

To further investigate the mechanism of VEGFA on TNBC cell stemness, we next examined the expression of the VEGFA receptor on TNBC cells. Previous studies have proven that VEGFA binds to VEGFR2 to stimulate angiogenesis. For NRP-1, studies have reported that the intracellular region contains only 40 amino acids and lacks intrinsic enzymatic activity^27^, which always acts as a coreceptor with VEGFR2 and other receptors^28^. Recent studies indicate that VEGFA seems to interact with NRP-1 alone and exert biological functions^27^. In our study, as shown in Figure 3A-C, NRP-1 was highly expressed in both MDA-MB-231 and SUM159 cells, while nearly no NRP-1 was detected in other molecular subtype cell lines, indicating that VEGFA may interact with NRP-1 and further activate intracellular signaling pathways in TNBC cells. To verify the impact of NRP-1, we next knocked down the expression of NRP-1 in MDA-MB-231 and SUM159 cells (Figure S1G-J). Knockdown of NRP-1 markedly reduced microsphere formation and impeded the expression of the CSC markers CD44, OCT-4, Nanog and SOX-2 (Figure 3D-H). Moreover, knockdown of NRP-1 markedly attenuated TNBC cell migration and invasion *in vitro* (Figure S3A-H). Notably, this inhibition could not be reversed after treatment with exogenous hVEGF_165_. This suggests that NRP-1 is required for VEGFA action on MDA-MB-231 and SUM159 cells. Altogether, these results reveal that VEGFA is highly expressed in TAMs and TNBC cells and promotes the CSC phenotype via NRP-1 in TNBC cells.

### GAPVD1 interacts with NRP-1 and mediates the effect of the VEGFA/NRP-1 axis in TNBC

**Figure 4:**
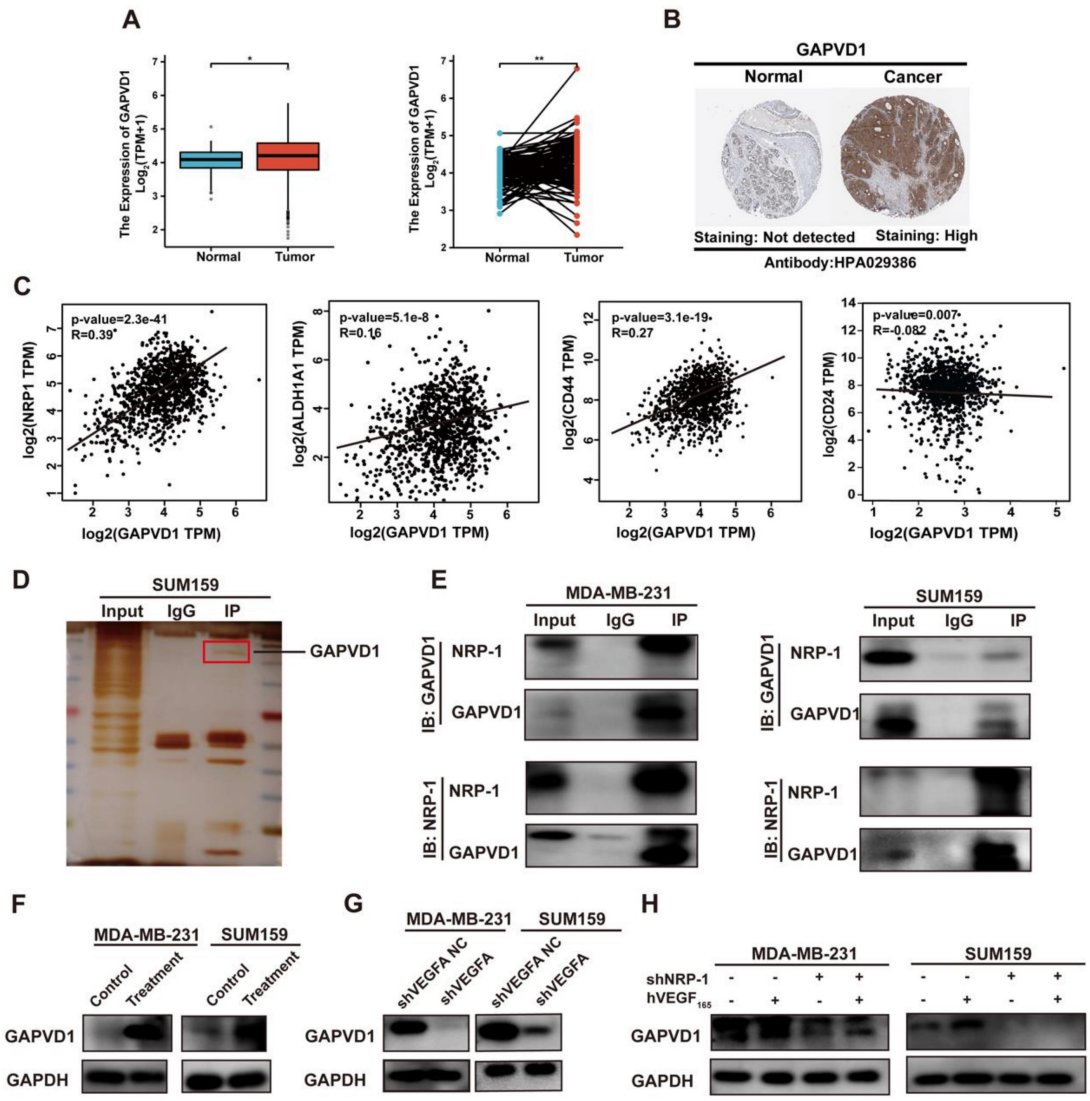
** GAPVD1 interacts with NRP-1 and is regulated by the VEGFA/NRP-1 axis. (A)** GAPVD1 mRNA expression in nonpaired (left panel, adjacent noncancerous tissue n=113, cancer tissue n=1113) and paired (right panel, n=113) breast cancer samples from the TCGA database. The error bar indicates the mean ± SD. **(B)** GAPVD1 protein expression in breast cancer tissue from the HPA database. **(C)** Correlation between GAPVD1 and NRP-1, ALDH1, CD44, and CD24 mRNA expression in human breast cancer samples from the TCGA dataset. **(D-E)** Coimmunoprecipitation of NRP-1 with the GAPVD1 antibody from TNBC whole-cell extracts. Precipitation with normal rabbit IgG was used as a negative control. **(F)** Western blotting of GAPVD1 after treatment with 10 ng/ml hVEGF_165_ in TNBC cells. **(G)** Western blotting of GAPVD1 after VEGFA knockdown in TNBC cells. **(H)** Western blotting of GAPVD1 after NRP-1 knockdown in the presence or absence of 10 ng/ml hVEGF_165_. The images show representative data, and data are expressed as the mean ± SD of each group of cells from three separate experiments. n.s., no significance, *P < 0.05, **P < 0.01 vs. the controls.

To identify the intracellular NRP-1-interacting proteins in TNBC cells, coimmunoprecipitation coupled with mass spectrometry was applied. Multiple analyses of the mass spectrometry results were performed to identify downstream targets of NRP-1. The mass spectrometry results revealed 880 proteins that bound uniquely to NRP-1 (Table S4). We first selected the top 10 best-scored target proteins and analyzed correlations between target proteins and NRP-1 and CSC markers, such as CD24, CD44 and ALDH1 (Figure S4, Table S5). A small GTPase regulator named GTPase-activating protein and VPS9 domain-containing protein 1, also known as GAPVD1, was identified as one of the highest-scoring proteins (Figure S5). We next analyzed GAPVD1 expression in human breast cancer via the TCGA and HPA databases. Figure 4A-B shows that GAPVD1 RNA and protein were highly expressed in breast cancer specimens compared to normal breast tissues. A strong correlation between GAPVD1 and NRP-1, CD24, CD44, and ALDH1 was displayed in the GEPIA database (Figure 4C). These findings suggest that GAPVD1 might be involved in regulating cancer stemness. We next performed Co-IP to validate the mass spectrometry results. Figure 4D-E shows NRP-1 and GAPVD1 coprecipitated with each other, and the interactions were specific, as no coprecipitation was observed when nonspecific immunoglobulin was utilized. This suggested a strong interaction between NRP-1 and GAPVD1 in TNBC cells.

To verify the role of GAPVD1 in the VEGFA/NRP-1 axis, we next detected the alteration of GAPVD1 after VEGFA/NRP-1 activation or inhibition. Western blotting validated that the expression of GAPVD1 was significantly increased upon treatment with exogenous hVEGF_165_ but decreased upon VEGFA or NRP-1 knockdown (Figure 4F-H). Notably, the inhibition of GAPVD1 expression upon NRP-1 knockdown could not be rescued by exogenous hVEGF_165_, indicating that the action of VEGFA on GAPVD1 was NRP-1 dependent.

### The VEGFA/NRP-1 axis promotes TNBC cell progression and stemness via GAPVD1

**Figure 5:**
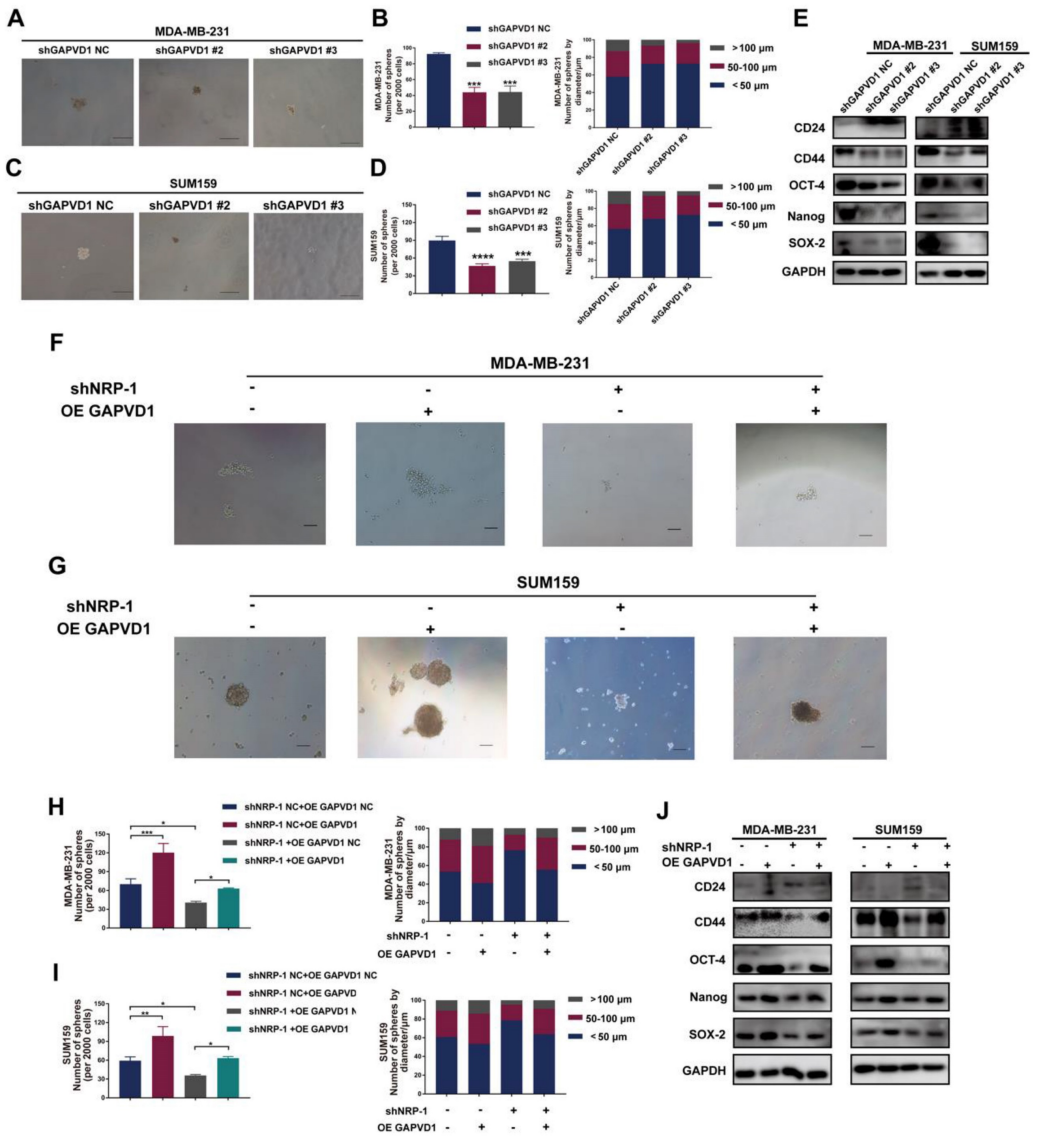
** The VEGFA/NRP-1 axis promotes TNBC cell progression and stemness via GAPVD1. (A-D)** Representative images of microspheres after GAPVD1 knockdown (shGAPVD1#2 and shGAPVD1#3) in TNBC cells. The number of microspheres was counted and plotted, and the percentage of microspheres with diameters of < 50 μm, 50-100 μm and > 100 μm was calculated and plotted (200 ×, scale bars = 100 μm). **(E)** Western blotting analysis of breast cancer stem cell markers (CD24, CD44, OCT-4, Nanog and SOX-2) in TNBC cells after GAPVD1 knockdown. **(F-I)** Representative images of microspheres after GAPVD1 overexpression (OE GAPVD1) in control and NRP-1-silencing TNBC cells. The number of microspheres was counted and plotted, and the percentage of microspheres with diameters of < 50 μm, 50-100 μm and > 100 μm was calculated and plotted (200 ×, scale bars = 100 μm). **(J)** Western blotting analysis of breast cancer stem cell markers (CD24, CD44, OCT-4, Nanog and SOX-2) after GAPVD1 overexpression in control and NRP-1-silencing TNBC cells. The images show representative data, and data are expressed as the mean ± SD of each group of cells from three separate experiments. *P < 0.05, **P < 0.01, ***P < 0.001, ****P < 0.0001 vs. the controls.

To determine whether the VEGFA/NRP-1-induced CSC phenotype is regulated via GAPVD1 in TNBC cells, we generated GAPVD1-knockdown and GAPVD1-overexpressing TNBC cells (Figure S6). Similar to VEGFA/NRP-1 inhibition, knockdown of GAPVD1 also reduced microsphere formation and cell mobility and impeded the expression of the CSC markers CD44, OCT-4, Nanog, and SOX-2, suggesting inhibition of the CSC phenotype (Figure 5A-E, S7A-H). Conversely, overexpression of GAPVD1 promoted microsphere formation, the expression of CSC markers and cell motility (Figure 5F-J, S7I-P). Most importantly, GAPVD1 overexpression rescued the NRP-1 inhibition-mediated reduction in CD44, OCT-4, Nanog and SOX-2 and rescued microsphere formation and mobility in TNBC cells (Figure 5F-J, S7I-P). These findings indicated that the VEGFA/NRP-1-induced CSC phenotype was enforced via the activation of GAPVD1. GAPVD1 acted as a downstream target in the VEGFA/NRP-1 axis.

### The VEGFA/NRP-1/GAPVD1 axis targets Wnt/β-catenin signaling in the regulation of the CSC phenotype in TNBC cells

**Figure 6:**
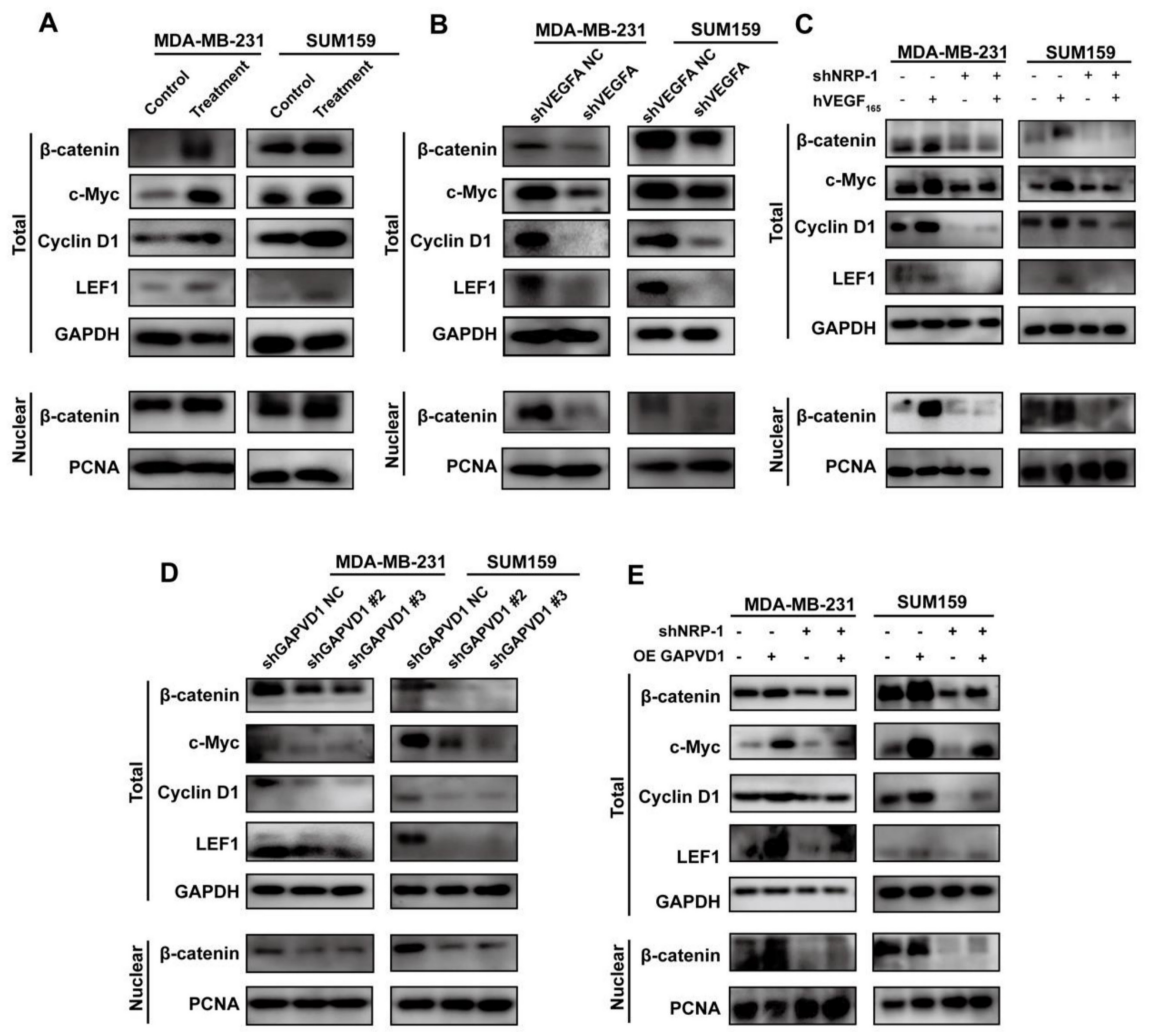
** The VEGFA/NRP-1/GAPVD1 axis targeted the downstream Wnt/β-catenin signaling pathway. (A)** Western blotting analysis of β-catenin and Wnt/β-catenin downstream targets in TNBC cells after treatment with 10 ng/ml hVEGF_165_. **(B)** Western blotting analysis of β-catenin and Wnt/β-catenin downstream targets in TNBC cells after VEGFA knockdown (shVEGFA). **(C)** Western blotting analysis of β-catenin and Wnt/β-catenin downstream targets in TNBC cells after NRP-1 knockdown (shNRP-1) in the presence or absence of 10 ng/ml hVEGF_165_. **(D)** Western blotting analysis of β-catenin and Wnt/β-catenin downstream targets in TNBC cells after GAPVD1 knockdown (shGAPVD1#2 and shGAPVD1#3). **(E)** Western blotting analysis of β-catenin and Wnt/β-catenin downstream targets in control and NRP-1-silencing TNBC cells after GAPVD1 overexpression (OE GAPVD1). The images show representative data, and data are expressed as the mean ± SD of each group of cells from three separate experiments. *P < 0.05, **P < 0.01, ****P < 0.0001 vs. the controls.

We have described that VEGFA was highly expressed in TNBC cells and promoted the CSC phenotype via the NRP-1/GAPVD1 axis. GAPVD1 was previously reported as a GTPase regulator, but the precise mechanism of cancer stemness in TNBC cells remains unclear. Wnt/β-catenin signaling plays an important role in the self-renewal and regulation of cancer stemness in many types of malignancies 29. We next detected the expression of β-catenin and its target genes c-Myc, CyclinD1 and LEF1 after alteration of the VEGFA/NRP-1/GAPVD1 axis. As shown in Figure 6A, treatment with exogenous hVEGF_165_ significantly increased the expression of β-catenin, c-Myc, CyclinD1 and LEF1. VEGFA/NRP-1 knockdown demonstrated the opposite effect on β-catenin and its target genes (Figure 6B-C). Additionally, nuclear β-catenin expression was markedly decreased after VEGFA/NRP-1 knockdown, indicating decreased transcriptional activity of Wnt/β-catenin signaling. Similarly, decreased expression of GAPVD1 resulted in the same effect on β-catenin, c-Myc, CyclinD1 and LEF1 (Figure 6D). GAPVD1 overexpression successfully restored the expression of β-catenin and its target genes upon NRP-1 knockdown (Figure 6E). These data suggested that the VEGFA/NRP-1/GAPVD1 axis might regulate the TNBC cell CSC phenotype by targeting the Wnt/β-catenin signaling pathway.

### Inhibition of GAPVD1 impeded tumor growth and cancer stemness *in vivo,* and GAPVD1 expression indicated poor prognosis in TNBC

**Figure 7:**
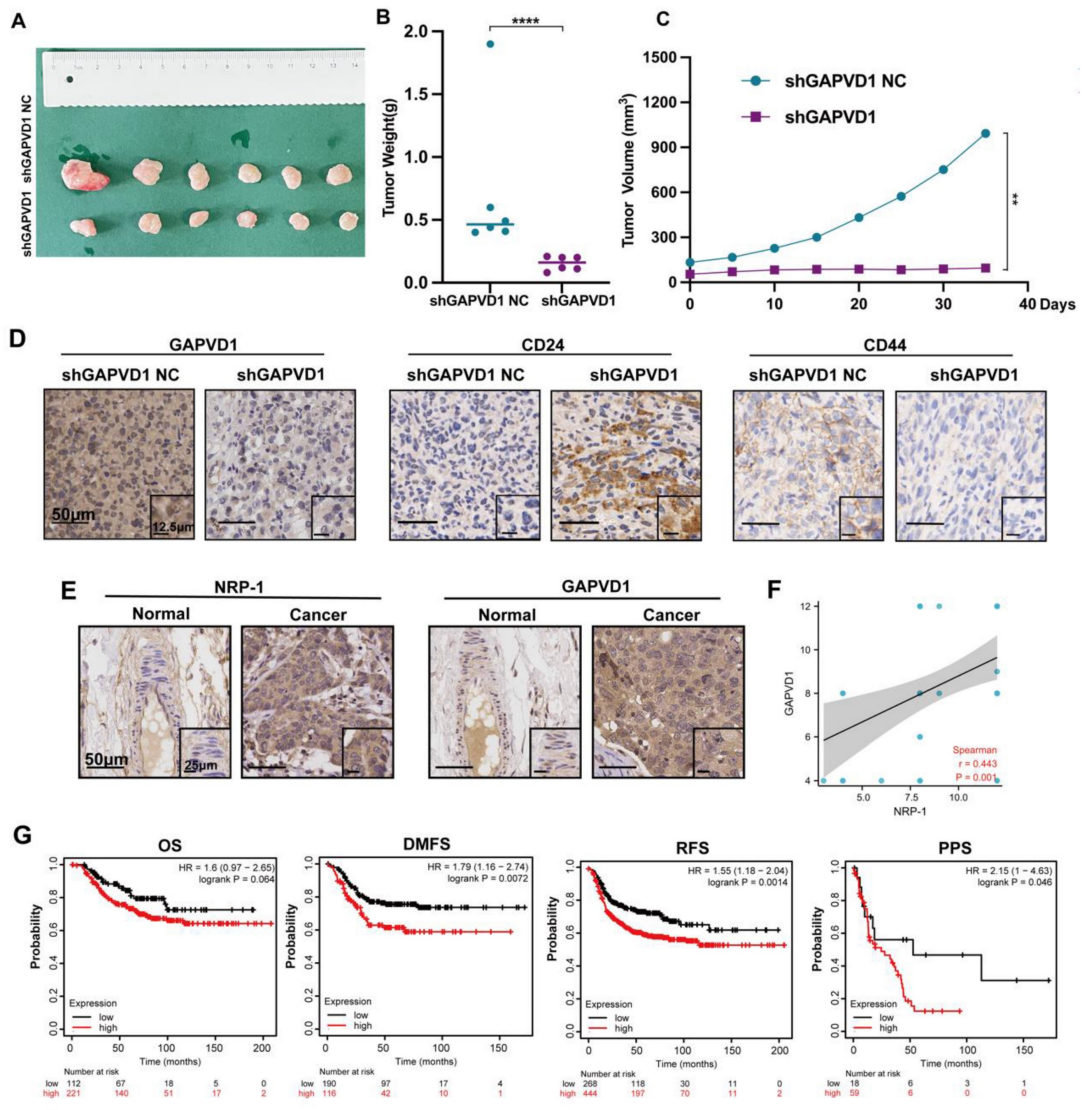
** GAPVD1 inhibition impedes tumor growth and cancer stemness *in vivo,* and a high level of GAPVD1 indicates a poor prognosis in TNBC. (A)** Diagram of xenograft tumors in NOD/SCID mice. Female NOD/SCID mice were randomized and inoculated with MDA-MB-231/shGAPVD1 NC (control group) or MDA-MB-231/shGAPVD1 cells (GAPVD1 knockdown group), and the growth of implanted breast tumors was monitored. **(B)** Tumor weight in the indicated groups. **(C)** The growth curve of inoculated breast tumors. **(D)** Representative IHC images of GAPVD1 and stem cell marker (CD24 and CD44) expression in xenograft tumors. **(E)** Representative IHC images of NRP-1 and GAPVD1 in a tissue microarray (adjacent noncancerous breast specimens n=5, TNBC specimens n=50). **(F)** Correlation analysis of NRP-1 and GAPVD1 expression in tissue microarray specimens. **(G)** Kaplan‒Meier analysis of the OS (high n=221, low n=112), DMFS (high n=116, low n=190), RFS (high n=444, low n=268) and PPS (high n=59, low n=18) of TNBC patients displaying high or low GAPVD1 expression. The images show representative data, and data are expressed as the mean ± SD of each group. **P < 0.01, ****P < 0.0001 vs. the controls.

Our* in vitro* data suggested that GAPVD1 was the downstream mediator of the VEGFA/NRP-1 axis, and upregulation of GAPVD1 abrogated the effect of VEGFA and NRP-1 inhibition. Next, we assessed the effect of tumor growth and stem cell phenotype *in vivo* after GAPVD1 blockade. Knockdown of GAPVD1 significantly attenuated tumor growth in NOD/SCID mice (Figure 7A-C). Immunohistochemistry revealed attenuated CD44 expression and elevated CD24 expression in the GAPVD1 knockdown group *in vivo* (Figure 7D). Our *in vitro* and *in vivo* experiments showed that GAPVD1 acted as a downstream effector of the VEGF/NRP-1 axis. To verify its potential role in clinical practice, we next analyzed the expression and prognostic value of GAPVD1 in human TNBC tissues. Figure 7E-F shows that GAPVD1 was highly expressed in TNBC specimens compared to normal breast tissue. High GAPVD1 and NRP-1 expression correlated with lymph node metastasis and tumor size in TNBC (Table S6). In addition, a high level of GAPVD1 indicated shorter DMFS, RFS and PPS in TNBC patients (Figure 7G). These findings implicated GAPVD1involved in the regulation of cancer stemness *in vivo,* and high expression of GAPVD1 predicted a poor prognosis in TNBC.

### TNBC cells-derived VEGFA promotes TAMs polarization into M2 type and reciprocally constitute a feedback loop

**Figure 8:**
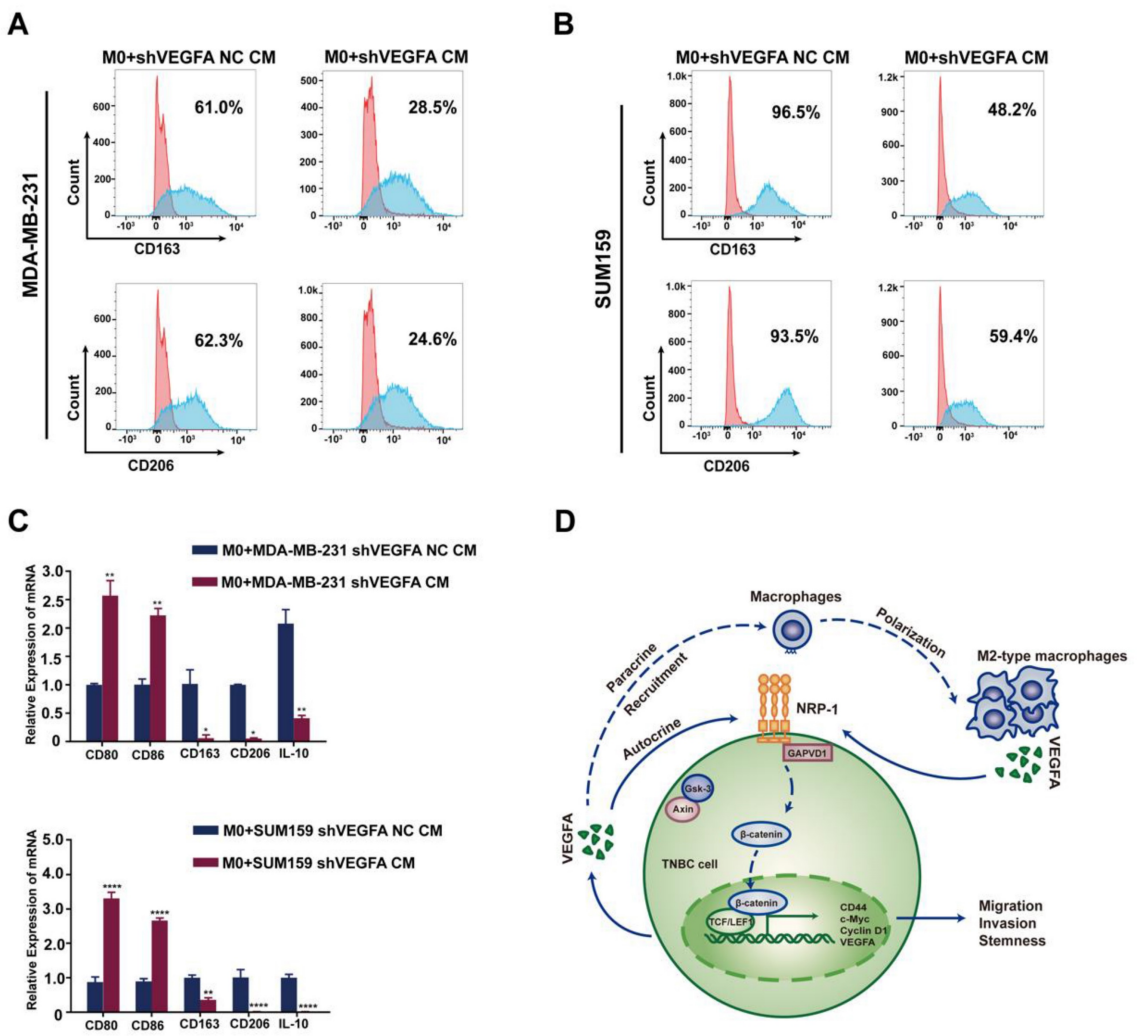
** TNBC cell-derived VEGFA promotes TAM polarization into M2 type and schematic of the crosstalk between TAMs and TNBC cells. (A-B)** Flow cytometry analysis of the expressions of M2 macrophage markers (CD163 and CD206) after co-cultured with conditional medium from control TNBC cells or VEGFA-silencing TNBC cells. **(C)** RT-qPCR analysis of M1-related markers (CD80 and CD86) and M2-related markers (CD163, CD206 and IL-10) expression after co-cultured with conditional medium from control TNBC cells or VEGFA-silencing TNBC cells. The images show representative data, and data are expressed as the mean ± SD of each group of cells from three separate experiments. *P < 0.05, **P < 0.01, ****P < 0.0001 vs. the controls. **(D)** Schematic of the crosstalk between TAMs and TNBC cells mediated by VEGFA in the promotion of breast cancer stemness. VEGFA (secreted by TAMs in a paracrine manner and by TNBC cells in an autocrine manner) binds to NRP-1 and activates the downstream GAPVD1/Wnt/β-catenin signaling pathway to promote the stemness of TNBC. Additionally, VEGFA may provide an immunosuppressive microenvironment for tumor progression by recruiting TAMs and facilitating the M2 polarization of TAMs.

Macrophages exhibit diversity of functions and plasticity which could be educated by tumor cells. Thus we speculate TNBC cells may in turn regulate the polarization and function of macrophages. When cocultured with conditional medium of TNBC cells, a shift of M2 polarization was observed in tumor-associated macrophages characteristic by the elevated expression of CD206 and CD163. While cocultured with the conditional medium of VEGFA knockdown TNBC cells, this polarization was apparently attenuated (Figure 8A-C). Taken together, these data preliminary indicated tumor cell-derived VEGFA participated in polarization of macrophages and contributed to the formation of immune supression microenvironment in TNBC. As to VEGFA, we showed that VEGFA produced by TAMs promoted the cancer stemness of TNBC cells via the NRP-1 receptor and downstream GAPVD1/Wnt/β-catenin signaling pathway. Meanwhile, tumor cell-derived VEGFA devoted to polarization of macrophages. There may be a feed-back loop mediated by VEGFA between TNBC cells and macrophages and this process could be amplified in the development of cancers (Figure 8D). Targeted this process may provide new opinion on the cancer therapy of TNBC.

## Discussion

TNBC is the most aggressive subtype of breast cancer with a high incidence of mortality and lacks an effective therapeutic target 32. Recent studies suggest that VEGFA/NRP-1 acts as an important effector in regulating the CSC phenotype and TME 23, 33. However, the precise role and mechanism remain unclear. In the present study, we report that M2-type TAMs promote the cancer stemness of TNBC cells through the secretion of VEGFA*.* VEGFA facilitates the CSC phenotype via NRP-1 and the downstream GAPVD1/Wnt/β-catenin signaling pathway. Reciprocally, tumor cell-derived VEGFA contributes to M2 polarization of macrophages in TNBC. Our findings document an interactive dialog between TNBC cells and TAMs implemented by VEGFA. VEGFA maintains cancer stemness and progression of TNBC through the NRP-1/GAPVD1 axis and Wnt/β-catenin signaling pathway. These findings suggest that TAM-targeted immunotherapy and VEGFA-targeted therapy could be combined for TNBC treatment.

TNBC is characterized by a unique TME that differs from that of other subtypes 34, 35. TAMs are the main component of the TME and demonstrate two functional states. M2 macrophages, which are defined as the protumorigenic state, play dramatically significant roles in tumor initiation and progression 36. Zhang *et al.* 37 reported that hypoxic pressure promoted M2 macrophage generation and further promoted cancer progression and temozolomide (TMZ) resistance in GBM by secreting VEGF. Another study identified that TAM-derived TGF-β1 induced ferroptosis resistance in TNBC cells and hence enhanced progression and chemoresistance by regulating the SMAD3/HLF/GGT1/GPX4 pathway 9. Wang *et al.* 38 showed that M2 macrophages induced by hypoxic exosomes promoted the migration, invasion and EMT of pancreatic cancer cells. In this study, we found that M2-type TAMs expressed high levels of VEGFA and promoted the migration, invasion and cancer stemness of TNBC cells by secreting VEGFA. Knockdown of VEGFA expression in M2-type TAMs attenuated migration, invasion and CSC marker expression in TNBC cells. Notably, the use of exogenous hVEGF_165_ to stimulate the invasiveness and stem cell marker expression of VEGFA-silencing TAMs further supported the importance of VEGFA in TAM-mediated signaling and function. Altogether, these results indicated that M2-type TAMs induces TNBC progression via VEGFA.

In addition to its roles in angiogenesis, VEGFA has also been implicated in regulating cancer cell survival, migration, invasion and EMT 10, 11. Recently, accumulated evidence has indicated that VEGFA may be involved in regulating the CSC phenotype. Angom *et al.* 23 reported that patient-derived GBM cells expressing shRNAs of VEGF or NRP-1 attenuated CSC markers and inhibited the neurosphere-forming capacity and migration of tumor-initiating cells. Grun *et al.* 22 found that VEGFA acted via NRP-1 to enhance epidermal CSC survival and the formation of aggressive and highly vascularized tumors. Mathilde *et al.* 39 found that NRP-1 modulated the 3D invasive properties of glioblastoma stem-like cells, and both RNA interference-mediated silencing and CRISPR-mediated gene editing deletion of NRP-1 strongly impaired the 3D invasive properties of patient-derived cells with stem-like properties and their close localization to brain blood vessels. Another study reported that NRP-1 was overexpressed in medulloblastoma (MB) and related to the undifferentiated status of MB and that an NRP-1 inhibitor (MR438) could stimulate the differentiation of MB stem-like cells. Targeting NRP-1 with MR438 could limit MB progression by decreasing the stem cell number while reducing the radiation dose 40, 41. These studies suggest that the VEGFA/NRP-1 axis plays an important role in the formation and maintenance of CSC features in several malignancies. However, the precise role of this axis in CSCs of TNBC remains unclear. In the present study, we find that VEGFA promotes the formation and maintenance of the CSC phenotype in TNBC cells via the NRP-1 receptor. The failure of exogenous hVEGF_165_ to stimulate the invasiveness and sphere formation of NRP-1-silencing breast cancer cells further supported the importance of NRP-1 in VEGFA-mediated signaling and function. Therefore, we attempted to identify the molecular entity that mediates VEGFA/NRP-1 action.

NRP-1 contains only a small 40-amino acid intracellular domain and lacks intrinsic kinase activity, it was initially thought that NRP-1 functions as a coreceptor with VEGFR2 and other receptors^42^, ^43^. Recently, several studies indicated that VEGFA interacts with NRP-1 via novel signaling pathways that are independent of other VEGF receptors to activate cellular processes^22^. However, the specific mechanism of downstream messengers and signaling pathways needs to be further elucidated. In the current study, through Co-IP coupled with MS, a small GTPase regulator named GAPVD1 was identified as a target downstream of NRP-1. We found a strong interaction between NRP-1 and GAPVD1 in TNBC cells. Further study revealed that the VEGFA/NRP-1-induced CSC phenotype was facilitated via its regulation of GAPVD1 in TNBC cells. In addition, we also demonstrated that knockdown of GAPVD1 significantly attenuated tumor growth in NOD/SCID mice *in vivo* and that GAPVD1 expression indicated poor prognosis in TNBC specimens. Collectively, these data showed that the VEGFA/NRP-1/GAPVD1 axis is involved in the regulation of cancer stemness and mobility in TNBC cells and that GAPVD1 acts as an effector downstream of NRP-1 in this process. However, the exact interaction mode of NRP-1 and GAPVD1 was not elucidated in this study. A conserved PDZ domain-binding motif (SEA) at the c-terminus of NRP-1 was found to bind to the GAIP interacting protein C-terminus/synectin, which mediates intracellular signaling and receptor internalization^42^. Yaqoob *et al.* showed that NRP-1 promoted integrin function both by binding fibronectin and by activating the intracellular kinase c-Abl through coordinated actions of both its intracellular and extracellular protein domains, and c-Abl promoted the function of integrin family members by activating small GTPases such as Rac or Rho^44^. Thus, in TNBC cells, it is possible that NRP-1 interacts with the small GTPase GAPVD1 in a similar manner. Apparently, our study extended previous observations that the VEGFA/NRP-1 axis is involved in the regulation of tumorigenesis and cancer stemness of breast cancer through GAPVD1, and these findings further broaden the notion that the VEGFA/NRP-1/GAPVD1 axis may be a valuable therapeutic target for the intervention of TNBC.

The Wnt/β-catenin signaling pathway has been well studied for its essential function in development and CSC biology. Reports have proven that numerous proto-oncogenes stimulate self-renewal of the CSC phenotype through the Wnt/β-catenin signaling pathway^45^. In the current study, we explored the expression of Wnt/β-catenin signaling and its target genes after modulation of the VEGFA/NRP-1/GAPVD1 axis. Inhibition of the VEGFA/NRP-1/GAPVD1 axis significantly impeded the expression of β-catenin, indicating repression of the Wnt/β-catenin pathway. Consistent with these data, the expression of c-Myc, Cyclin D1 and LEF1 was also decreased, supporting the findings that the Wnt/β-catenin pathway was inhibited after VEGFA/NRP-1/GAPVD1 axis blockade. In the shNRP-1 group, exogenous hVEGF_165_ failed to increase the expression levels of active β-catenin and the levels of c-Myc, Cyclin D1 and LEF1. Conversely, overexpression of GAPVD1 successfully overcame the effect of NRP-1 inhibition on the Wnt/β-catenin pathway. These two findings support that the function of VEGFA in TNBC cells is NRP-1 dependent and that the VEGFA/NRP-1 axis triggers the downstream pathway by modulating the expression of GAPVD1. Above all, our data support and broaden the concept that the VEGFA/NRP-1/GAPVD1 axis targets the downstream Wnt/β-catenin signaling pathway in regulating cancer stemness in TNBC cells.

CSCs colonize a specific tumor microenvironment and may educate several components in the stem cell niche 46. The literature indicates an important function for VEGFA in the regulation of the tumor immune microenvironment 47. A recent study indicated an immunosuppressive function of VEGFA directly driving T-cell exhaustion via TOX upregulation in the tumor microenvironment of MSS CRC 33. Huang *et al.* 48 found that EBV-replicating nasopharyngeal carcinoma cells successfully recruit monocytes and activate TAMs via VEGFA. The literature supports that tumor cell-derived VEGFA acts as a recruitment factor of macrophages 49. Our present results showed that VEGFA produced by TAMs promoted the cancer stemness of TNBC cells via the NRP-1 receptor and the downstream GAPVD1/Wnt/β-catenin signaling pathway. Also our data demonstrated TNBC cell-derived VEGFA induced a M2-type macrophage polarization. Thus, there may be a positive feedback loop mediated by VEGFA between TNBC cells and macrophages, and this process could be amplified in the development of cancers (Figure 8). Targeting this process may provide new options for TNBC treatment.

In summary, in the present study, we explored the reciprocal interaction between TNBC cells and TAMs mediated by VEGFA and further clarified the role and underlying mechanisms of VEGFA in regulating cancer stemness. Our study indicate that M2-type TAMs induce TNBC progression via the VEGFA/NRP-1 axis. Mechanistically, the VEGFA/NRP-1 axis triggers the downstream pathway by modulating the expression of GAPVD1 and the Wnt/β-catenin signaling pathway. Previous studies demonstrated that VEGFA acts as a chemoattractant in macrophage recruitment 48, 49. Our result demonstrates that tumor cell-derived VEGFA contributes to M2 polarization of macrophages in TNBC. Therefore, there may be a feedback loop mediated by VEGFA between TNBC cells and TAMs, and this process could be amplified in the development of cancers. Our findings support the potential application of combination regimens including immunotherapy and VEGFA-targeted agents in TNBC treatment. We recognize that our study has limitations. First, although we found that NRP-1 interacts with GAPVD1 in regulating TNBC cell stemness, the exact interaction mode of NRP-1 and GAPVD1 was not elucidated in this study. Second, our data did not exclude the effect of VEGFR2 in this process, and we are interested in further investigating the precise mechanisms underlying VEGFR/NRP-1-related signaling in breast cancer cells.

In conclusion, our data suggest that VEGFA acts as a hotspot in the reciprocal crosstalk between tumor cells and TAMs. TAM-derived VEGFA may promote cancer stemness of TNBC through the NRP-1/GAPVD1/Wnt/β-catenin axis in an autocrine and paracrine manner. Hence, the VEGFA/NRP-1/GAPVD1 axis may be a valuable target for TNBC therapy.

## Materials and Methods

### Bioinformatics analysis

The Gene Expression Omnibus (GEO), The Cancer Genome Atlas (TCGA), Cancer Cell Line Encyclopedia (CCLE) database, Gene Expression Profiling Interactive Analysis (GEPIA) database and Human Protein Atlas database were explored to confirm gene expression in breast cancer. Survival curves were drawn by Kaplan‒Meier plotter.

### Reagents and antibodies

hVEGF_165_ (Cell Signaling Technology, MA, USA) was diluted with serum-free medium. Antibodies against the following proteins were used: GAPDH (1:1000, Santa Cruz, USA), VEGFA (1:1000, Santa Cruz Biotechnology. Santa Cruz, USA), NRP-1 (1:1000, Abcam, Cambridge, United Kingdom), Nanog (1:2000, Proteintech, Chicago, USA), c-Myc (1:1000, Proteintech, Chicago, USA), CD44 (1:1000, Proteintech, Chicago, USA), CD24 (1:500, Proteintech, Chicago, USA), SOX2 (1:1000, Proteintech, Chicago, USA), OCT-4 (1:1000, Proteintech, Chicago, USA), LEF1 (1:1000, Santa Cruz Biotechnology, Santa Cruz, USA), Cyclin D1 (1:1000, Santa Cruz Biotechnology, Santa Cruz, USA), β-catenin (1:1000, Proteintech, Chicago, USA), PCNA (1:1000, Proteintech, Chicago, USA), GAPVD1 (1:5000, Bethyl Laboratories, Texas, USA) and horseradish peroxidase (HRP)-conjugated secondary antibody (1:2000, Beyotime Biotechnology, China). Ammonium bicarbonate (Sigma‒Aldrich, St. Louis, USA), dithiothreitol (DTT) (Sigma‒Aldrich, St. Louis, USA), iodoacetamide (IAA) (Sigma‒Aldrich, St. Louis, USA), sodium carbonate (Sigma‒Aldrich, St. Louis, USA), acetonitrile (J. T. Baker, Phillipsburg, USA), urea (Bio-Rad, Hercules, CA), sodium dodecyl sulfate (SDS) (Bio-Rad, Hercules, CA) and trypsin (Promega, Madison, USA) were used.

### Cell lines and culture

The human breast cancer cell lines MDA-MB-231, SK-BR3, MCF-7, T47D, SUM159, and ZR-75, the mammary epithelial cell line MCF-10A and the monocytic cell line THP-1 were obtained from the Cell Bank of the Chinese Academy of Sciences (Shanghai, China). MDA-MB-231 cells were maintained in L-15 (HyClone, Utah, USA) medium. SK-BR3, MCF-7 and T47D cells were cultured in DMEM medium (GIBCO, CA, USA). SUM159 was cultured in DMEM/F12 medium (GIBCO, CA, USA). ZR-75 and THP-1 cells were cultured in RPMI-1640 medium. All the cells above were cultured in the presence of 10% fetal bovine serum (FBS, GEMINI, USA) with 100 U/mL penicillin G and 100 mg/mL streptomycin sulfate in a 37°C incubator with 5% CO_2_. MCF-10A cells were cultured in DMEM/F12 supplemented with 5% horse serum (GIBCO, CA, USA), 0.5 μg/ml hydrocortisone (Sigma, St Louis, USA), 10 μg/ml insulin (GIBCO, CA, USA) and 20 ng/ml recombinant human EGF (PeproTech, NJ, USA). Induction and differentiation of macrophages were performed according to the established method 25. Briefly, to generate M2-like macrophages, THP-1 cells were treated with PMA (200 ng/ml, Abcam, MA, USA) for 6 h and then cultured with IL-4 (20 ng/mL, PeproTech, US) and IL-13 (20 ng/mL, PeproTech, US) for an additional 18 hours.

### Wound healing assays

For the wound healing assay, the cells were first cultured to full confluence in 6-well plates. A 200 μl pipet tip was used to create a wound in the cell monolayer. Then, the cells were incubated with serum-free medium. Representative images were captured using digital microscopy at 0 h, 24 h and 48 h after injury.

### Transwell assays

For migration and invasion assays, 24-well plates and 8 µm Transwell inserts (Corning Life Science, NY, USA) were used. Briefly, 2×10^4^ cancer cells suspended in 100 µl serum-free medium were added to the top chambers (8 µm pore size, Corning, NY, USA), and conditional medium with relevant treatment was added to the bottom chamber. After 24 hours of culture, the cells remaining on the membrane surface of the top chamber were removed with a cotton swab. The cells that migrated onto the bottom surface of the top chambers were fixed with 4% paraformaldehyde, stained with 0.1% crystal violet solution and imaged under a light microscope. For the invasion assay, the upper chambers were precoated with Matrigel (diluted at 1:8, 50 μl/well, Corning, NY, USA) after 48 hours of culture. Three visual fields were randomly chosen to calculate the number of migrated and invaded cells.

### Mammosphere formation assay

Breast cancer cells were suspended in triplicate in ultralow-attachment 24-well plates (Corning, NY, USA) in serum-free DMEM/F12 (HyClone, Utah, USA) medium supplemented with 20 ng/mL epidermal growth factor (EGF, PeproTech, NJ, USA), 20 ng/ml bFGF (PeproTech, NJ, USA) and 1× B27 (GIBCO, CA, USA). Then, 0.2 ml of new media was added to the cultures every five days. The plate was incubated at 37°C with 5% CO_2_ for 14 days. The numbers of spheres were determined by microscopy using a microscope (Nikon) ×10 objective with phase contrast. Multicellular structures greater than 50 µm in diameter were considered spheres. The total number of spheres from each well was counted and represented as the average of 3 wells.

### Flow cytometry analysis

Cells were dissociated into single cells by trypsin, resuspended (1×10^6^ cells/ml) and incubated with running buffer or the indicated monoclonal antibodies for 30 min at 4°C (anti-CD44 conjugated to PE, anti-CD24 conjugated to APC, anti-CD163 conjugated to PE, and anti-CD206 conjugated to FITC) (eBioscience, USA and BioLegend, USA). Isotype-matched conjugated nonimmune antibodies were used as a negative control. Samples were analyzed using a flow cytometer (BD Biosciences) and processed using FlowJo software (FlowJo_V10).

### ELISA analysis

The levels of VEGFA in the supernatants of cultured cells were determined by ELISA using specific kits (Proteintech, Chicago, USA) according to the manufacturer's instructions. Briefly, breast cancer cells were cultured in 6-well culture plates in medium at 37°C for 48 h. The cultured supernatants were collected, and the levels of VEGFA in the supernatants were determined in triplicate by ELISA. Absorbance at 450 nm (and 540 nm as normalization background) was determined using a microplate reader (Multiskan FC; Thermo Scientific). The different concentrations of VEGFA were provided by the supplier and used to establish a standard curve to determine the VEGFA concentrations.

### Western blotting analysis

Cells derived from the monolayer were lysed with RIPA buffer (Beyotime Biotechnology, China) with protease inhibitor (Roche, Basel, Switzerland) on ice. The concentration of total protein was quantified using a BCA Protein Assay Kit according to the manufacturer's instructions. Nuclear protein was extracted using a Nuclear Protein Extraction Kit (Solarbio, China). Equivalent amounts of total cellular protein (50-100 μg/lane) extracted from the cells of interest were resolved by SDS‒PAGE and transferred onto PVDF membranes (Millipore, MA, USA). The membranes were blocked with 5% nonfat skim milk at 37°C, blotted with primary antibodies overnight at 4°C and probed with secondary antibodies for 1 hour at room temperature. Finally, the membranes were visualized and measured by an ECL system (Amersham, Sweden), followed by imaging.

### Quantitative real-time PCR analysis

Total RNA in different breast cancer cells was extracted using a Fast 200 reagent RNA isolation system (Feijie, Shanghai, China) and used for quantitative real-time PCR (RT‒qPCR) according to established procedures (Taraka, Kyoto, Japan). The sequences of the primers used for RT‒qPCR in this study are listed below:

GAPDH forward, 5′-GTCTCCTCTGACTTCAACAGCG-3′

GAPDH reverse, 5′- ACCACCCTGTTGCTGTAGCCAA-3′

VEGFA forward, 5′-AACTTTCTGCTGTCTTGG-3′

VEGFA reverse, 5′-ACTTCGTGATGATTCTGC-3′

NRP-1 forward, 5′-AGGACAGAG ACTGCAAGTATGAC-3′

NRP-1 reverse, 5′-AACATTCAGGACCTCTCTTGA-3′

GAPVD1 forward, 5′-GGTGCTACTTCTTTGGTGGCTG-3′

GAPVD1 reverse, 5′-TCTCCACCAAGGTCTGTTCCTG-3′

CD80 forward, 5′-CTCTTGGTGCTGGCTGGTCTTT-3′

CD80 reverse, 5′-GCCAGTAGATGCGAGTTTGTGC-3′

CD86 forward, 5′-CCATCAGCTTGTCTGTTTCATTCC-3′

CD86 reverse, 5′-GCTGTAATCCAAGGAATGTGGTC-3′

CD163 forward, 5′-CCAGAAGGAACTTGTAGCCACAG-3′

CD163 reverse, 5′-CAGGCACCAAGCGTTTTGAGCT-3′

CD206 forward, 5′-GTGGTCCTCCTGATTGTGATAG-3′

CD206 reverse, 5′- CACTTGTTCCTGGACTCAGATTA-3′

### Lentivirus production and cell transfection

The lentiviral vectors NRP-1 shRNA, VEGFA shRNA, GAPVD1 shRNA and nontargeting control were designed and synthesized by GeneChem (Shanghai, China). GAPVD1-overexpressing adenoviral vectors were designed and synthesized by HanBio (Shanghai, China). The specific sequences of the indicated shRNAs are presented as follows. Cells were infected with relevant lentiviral shRNA for each gene according to the manufacturer's protocol, and after puromycin selection, stably selected cells were used for the experiments.

VEGFA lentiviral shRNA sequence: GACAAGAAAATCCCTGTGGT;

NRP-1 lentiviral shRNA sequence: GACCCATACCAGAGAATTA;

GAPVD1 lentiviral shRNA sequence: GGAGAACACACAAAGTGTTAT (#1);

GCCACTTTACATGAGCCAATT (#2);

GCTCATTCAGAGGCTCAATGC (#3).

### Coimmunoprecipitation (Co-IP) assays, mass spectrometry (MS) and protein identification

Cells were lysed in RIPA containing phosphatase inhibitors and protease inhibitors. Lysates were incubated with the indicated primary antibody or IgG (as a negative control) at 4 °C overnight. The antibody-protein complexes were pulled down with protein A/G agarose beads (Santa Cruz Biotechnology. Santa Cruz, USA) and applied for Western blot analysis with NRP-1 antibody (1:30, Abcam, Cambridge, United Kingdom) and GAPVD1 antibody (2-5 μg/mg lysate, Bethyl Laboratories).

Afterward, the bound proteins were extracted from IP beads and digested into peptides for further LC‒MS analysis. LC‒MS/MS experiments were performed on a Q Exactive Plus mass spectrometer that was coupled to Easy nLC (Thermo Scientific). Then the MS data were analyzed using MaxQuant software version 1.6.0.16. MS data were searched against the UniProtKB database (36080 total entries, downloaded 08/14/2018).

### Nude mouse xenograft and transplanted models

The experimental protocols were approved by the Animal Care and Use Committee of Xi'an Jiaotong University. Four-week-old female NOD/SCID mice were purchased from GemPharmatech Laboratory Animal (Nanjing, China) and housed in a specific pathogen-free facility with free access to autoclaved water and food. Individual mice were randomized and injected orthotopically with 5×10^6^ cells into their left fat pad (n = 6 per group). The growth of implanted tumors was monitored every 5 days using a Vernier caliper up to 35 days postimplantation. The volume of tumors was calculated using the following formula: 1/2 × length × width^2^. The mice were sacrificed, and the tumors were harvested and weighed after 5 weeks. The tumor tissue sections were analyzed by immunohistochemistry using anti-GAPVD1, anti-CD24 and anti-CD44 antibodies, and the intensity of the indicated IHC staining was evaluated in a blinded manner.

### Tissue microarray (TMA), immunohistochemistry (IHC) and IHC evaluation

This study was approved by the Ethics Committee of the First Affiliated Hospital of Xi'an Jiaotong University. A tissue microarray of paraffin-embedded tissues, including tissue from 50 triple-negative breast tumor tissues, was purchased from Shanghai Biochip (Shaanxi Kexin Biotechnology Co., Ltd). Tissue was fixed in 4% paraformaldehyde, processed and sectioned according to established procedures. Then, IHC staining was performed on the TMA using NRP-1 (1:100, Abcam, Cambridge, United Kingdom), GAPVD1 (1:100, Abnova, Taipei, China), CD44 (1:200, Proteintech, Chicago, USA) and CD24 (1:100, Proteintech, Chicago, USA) antibodies. The integrated optical density (IOD) value was assessed by ImageJ software.

Quantitative scoring of the staining of complete tumor sections was evaluated by staining intensity and the percentage of positively stained cells 51. The intensity of the immunostaining was classified into 4 categories: 0 for no staining, 1 for light yellow, 2 for brown, and 3 for brown positive cells. The area of positive staining was scored as follows: 0 as 0%-5% of positive structures were present, 1 for 6%-25%, 2 for 26%-50%, 3 for 51%-75% and 4 for >76% of positive structures. Three visual fields were randomly selected from each slice. Finally, the product of the staining intensity and the percentage of positive cells was used as the result of immunohistochemistry. A score of 0 was negative, 1-4 was weakly positive, 5-8 was moderately positive, and 9-12 was strongly positive. We classified moderately positive and strongly positive as highly expressed and negative and weakly positive as weakly expressed for statistical analysis of the results.

### Statistical analysis

All data are presented as the mean ± SD from at least three independent experiments. Statistical analysis was performed by one-way ANOVA, two-way ANOVA or Fisher's exact test when applicable. All statistical analyses were performed using Prism 7.0 (GraphPad Software, La Jolla, CA). A *P* value of < 0.05 was considered to indicate statistical significance.

## Supplementary Material

Supplementary figures and tables.
