# Supplementary material for: VEGFA/NRP-1/GAPVD1 axis promotes progression and cancer stemness of triple-negative breast cancer by enhancing tumor cell-macrophage crosstalk
Source: Int J Biol Sci. 2024 Jan 1;20(2):446–63. doi: 10.7150/ijbs.86085 (PMC10758102; doi:10.7150/ijbs.86085)
Supplement: Supplementary file 1 — Supplementary figures and tables. [file ijbsv20p0446s1.zip › Supplementary materials/Supplemental Figures.pdf]

Figure S1

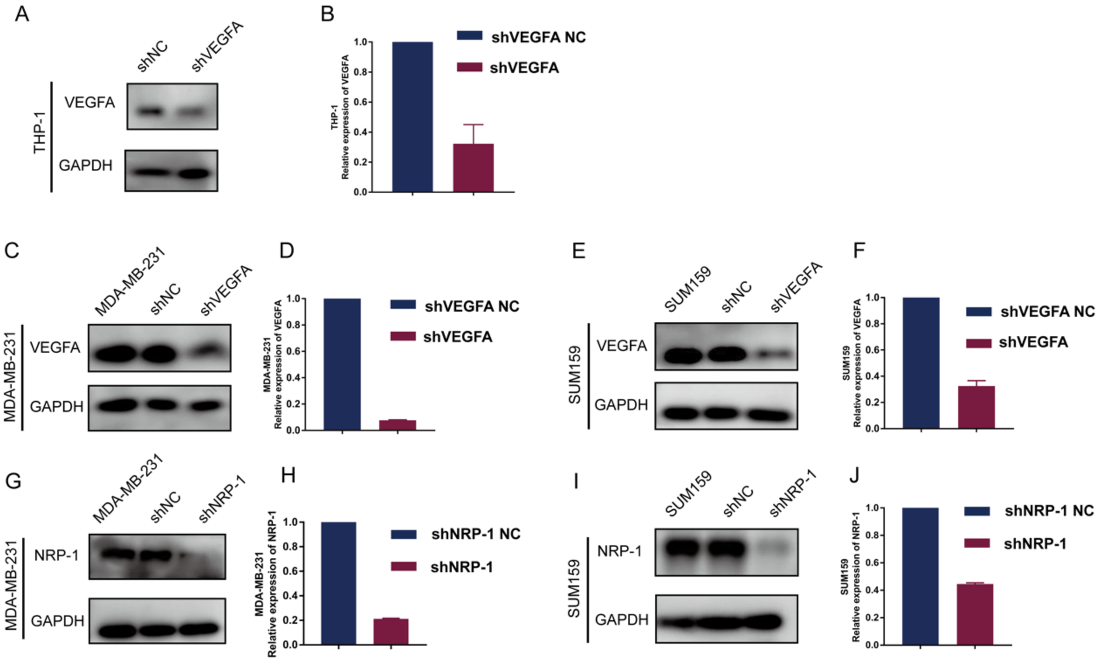

Figure S1 The efficiency of VEGFA and NRP-1 knockdown was verified by Western blotting and RT-qPCR

(A-B) RT-qPCR and Western blotting analysis of VEGFA knockdown efficiency in THP-1 cells. (C-F) RT-qPCR and Western blotting analysis of VEGFA knockdown efficiency in TNBC cells. (G-J) RT-qPCR and Western blotting analysis of NRP-1 knockdown efficiency in TNBC cells. Data are representative images and expressed as the mean ± SD of each group of cells from three separate experiments.

Figure S2

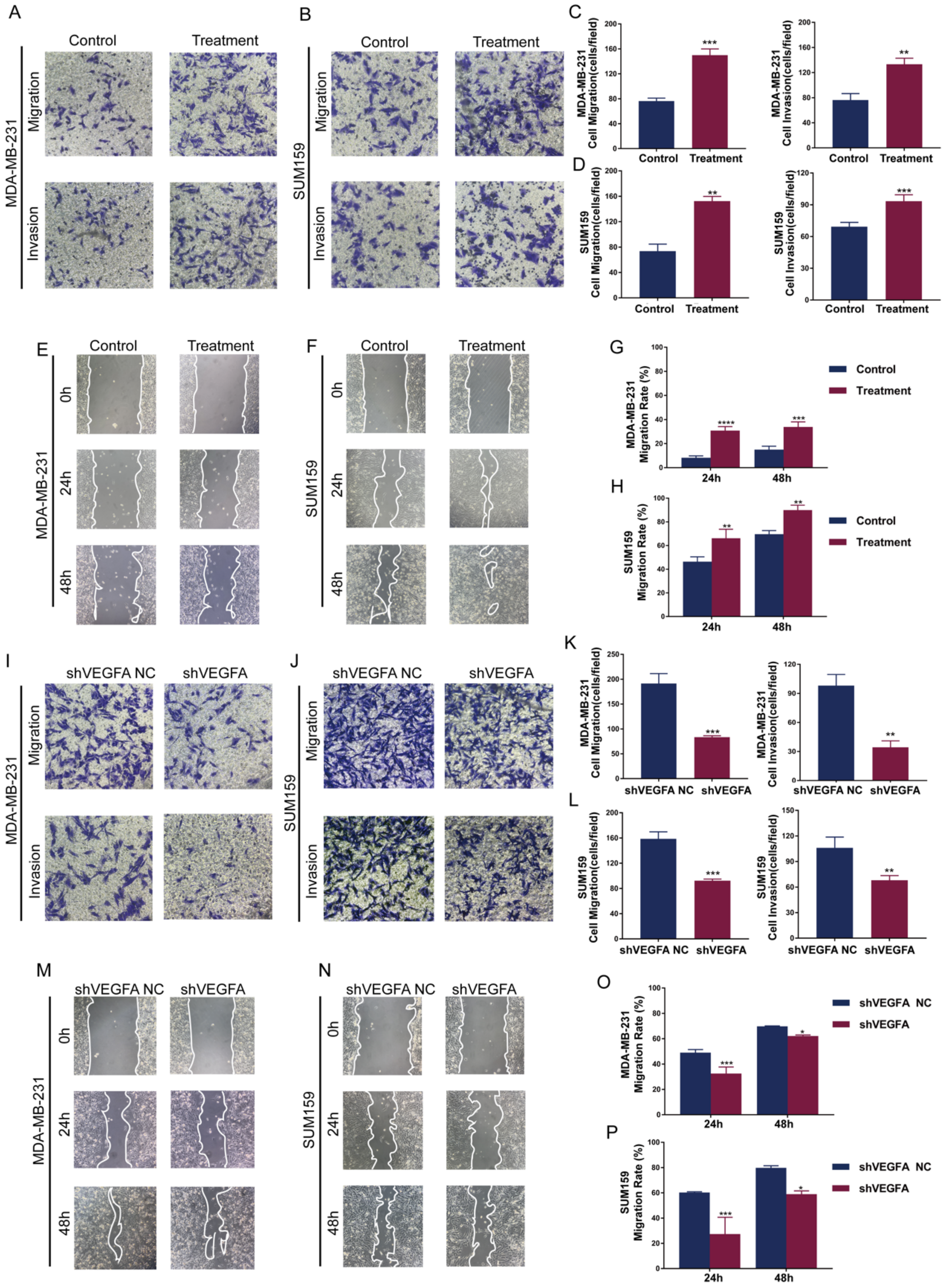

Figure S2 VEGFA regulates migration and invasion of TNBC cells

(A-H) The effect of exogenous hVEGFA$_{165}$ (10 ng/ml) on the migration and invasion of TNBC cells revealed by transwell and wound healing assay. (I-P) The effect of VEGFA knockdown on the migration and invasion of TNBC cells revealed by transwell and wound healing assay. Data are representative images and expressed as the mean ± SD of each group of cells from three separate experiments. *$P < 0.05$, **$P < 0.01$, ***$P < 0.001$, ****$P < 0.0001$ vs. the controls.

Figure S3

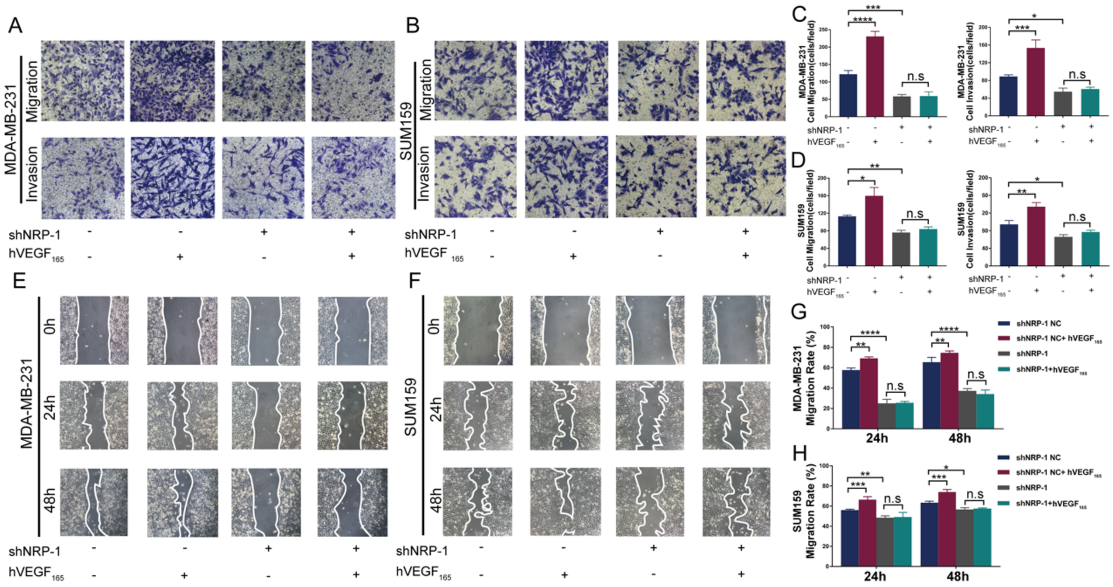

Figure S3 VEGFA modulates migration and invasion of TNBC cells in a NRP-1 dependent manner

(A-H) The effect of NRP-1 knockdown in the presence or absence of 10 ng/ml of $hVEGF_{165}$ on the migration and invasion of TNBC cells revealed by Transwell and wound healing assay. Data are representative images and expressed as the mean ± SD of each group of cells from three separate experiments. n.s no significance, $*P < 0.05$, $**P < 0.01$, $***P < 0.001$, $****P < 0.0001$ vs. the controls.

Figure S4

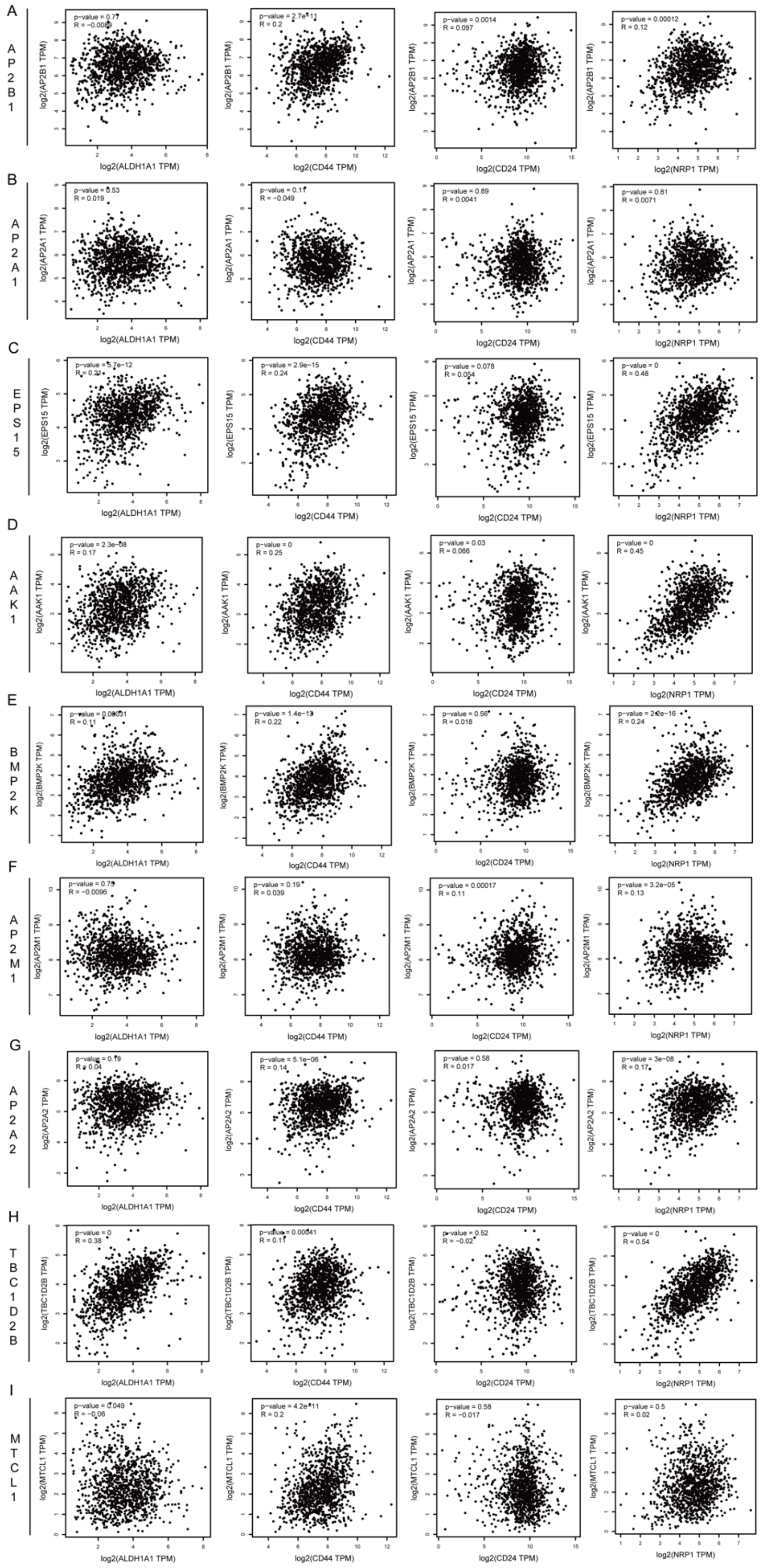

Figure S4 Correlations between MS target proteins and NRP-1 and cancer stem cell markers

(A-H) Correlations between MS target proteins and NRP-1 and cancer stem cell markers.

Figure S5

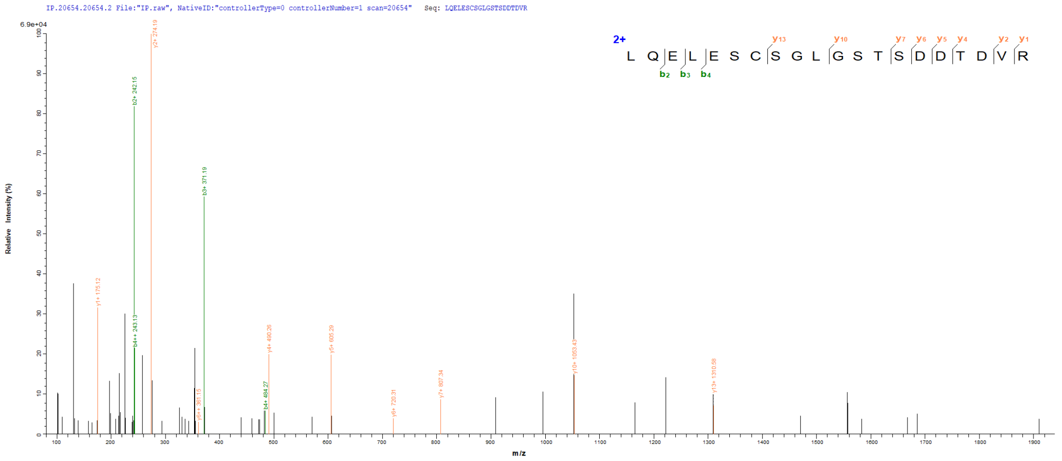

Figure S5 Identification of GAPVD1 peptide using LC-MS/MS

Figure S6

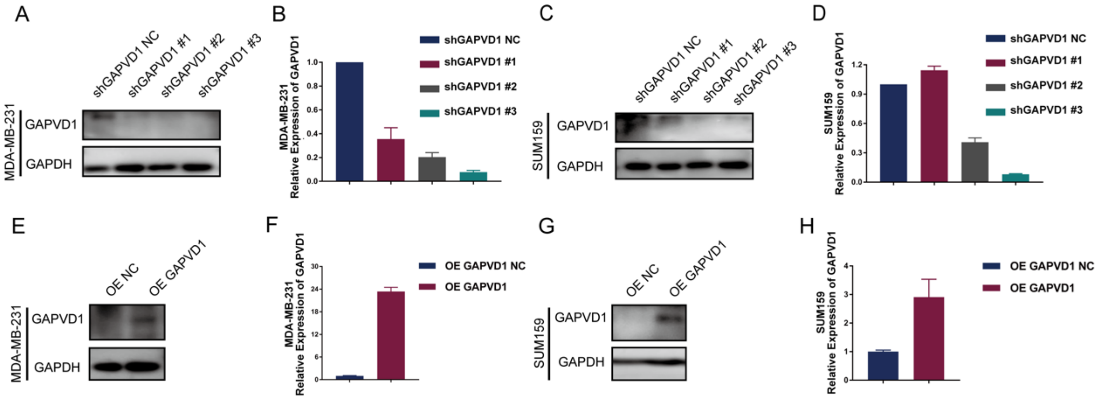

Figure S6 The efficiency of GAPVD1 silencing and overexpression was verified by Western blotting and RT-qPCR

(A-D) RT-qPCR and Western blotting analysis of GAPVD1 knockdown efficiency in TNBC cells, shRNA sequence #2 and #3 creating higher knockdown efficiency and were used for subsequent experiments. (E-H) RT-qPCR and Western blotting analysis of GAPVD1 overexpression efficiency in TNBC cells. Data are representative images and expressed as the mean ± SD of each group of cells from three separate experiments.

Figure S7

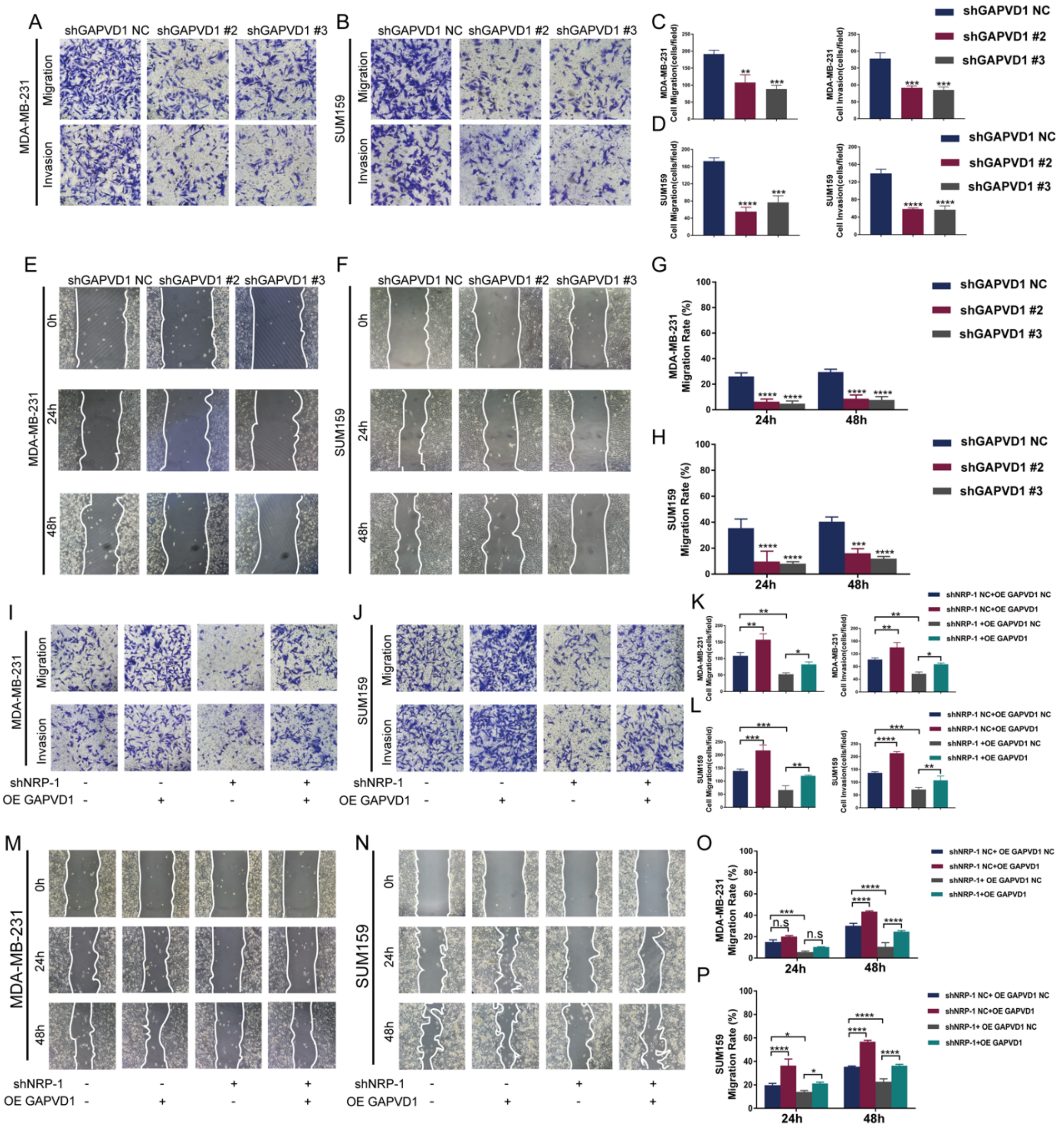

Figure S7 GAPVD1 regulates migration and invasion of TNBC cells

(A-H) The effect of GAPVD1 knockdown on the migration and invasion of TNBC cells revealed by Transwell and wound healing assay. (I-P) Migration and invasion revealed by Transwell and wound healing assay after GAPVD1 overexpression in control and NRP-1-silencing TNBC cells. Data are representative images and expressed as the mean ± SD of each group of cells from three separate experiments. n.s no significance, **P* < 0.05, ***P* < 0.01, ****P* < 0.001, *****P* < 0.0001 vs. the controls.

Table S3 The Overlap Genes Between GeneCard and GEO Dataset

Gene Symbol	Gene Title	adj.P.Value	logFC
IL1B	interleukin 1 beta	3.80E-07	1.10E+01
GDF15	growth differentiation factor 15	4.30E-07	9.87
IL1RN	interleukin 1 receptor antagonist	1.72E-06	5.38
S100A9	S100 calcium binding protein A9	3.62E-06	-4.55
HBEGF	heparin binding EGF like growth factor	3.98E-06	3.17
S100A8	S100 calcium binding protein A8	6.71E-06	-6.67
PPBP	pro-platelet basic protein	7.68E-06	6.36
FGF2	fibroblast growth factor 2	9.45E-06	4.57
ISM1	isthmin 1, angiogenesis inhibitor	1.27E-05	3.71
ANGPTL4	angiopoietin like 4	1.29E-05	4.91
CCL13	C-C motif chemokine ligand 13	1.44E-05	4.97
TNFSF10	tumor necrosis factor superfamily member 10	2.32E-05	-3.27
CCL2	C-C motif chemokine ligand 2	2.68E-05	5.35
EBI3	Epstein-Barr virus induced 3	2.77E-05	3.31
S100A11	S100 calcium binding protein A11	3.13E-05	2.08
AREG	amphiregulin	3.52E-05	7.48
CCL4	C-C motif chemokine ligand 4	4.11E-05	4.58
CCL7	C-C motif chemokine ligand 7	4.54E-05	7.27
PDGFA	platelet derived growth factor subunit A	4.97E-05	5.83
VEGFC	vascular endothelial growth factor C	5.31E-05	3.02
GDF11	growth differentiation factor 11	6.04E-05	-2.35
CCL8	C-C motif chemokine ligand 8	6.31E-05	5.7
VEGFA	vascular endothelial growth factor A	9.43E-05	3.06
CCL26	C-C motif chemokine ligand 26	1.06E-04	2.14
CCL3	C-C motif chemokine ligand 3	2.07E-04	2
BDNF	brain derived neurotrophic factor	2.38E-04	2.34
S100A10	S100 calcium binding protein A10	2.52E-04	2.31
WNT7B	Wnt family member 7B	2.70E-04	-2.23
INHBE	inhibin beta E subunit	2.86E-04	2.95
NRG1	neuregulin 1	2.87E-04	5.77
S100A12	S100 calcium binding protein A12	3.08E-04	-3.39
ANGPTL6	angiopoietin like 6	3.20E-04	2.04
CCL20	C-C motif chemokine ligand 20	3.34E-04	8.3
TNFSF15	tumor necrosis factor superfamily member 15	3.54E-04	4.4
CXCL8	C-X-C motif chemokine ligand 8	3.65E-04	8.91
TGFB3	transforming growth factor beta 3	4.02E-04	3.61
FST	follistatin	5.03E-04	2.39
IL36G	interleukin 36, gamma	5.23E-04	3.77
TCHH	trichohyalin	5.96E-04	-2.93
TNFSF13B	tumor necrosis factor superfamily member 13b	7.47E-04	-2.28
BMP8A	bone morphogenetic protein 8a	7.71E-04	2.4

IL1A	interleukin 1 alpha	1.78E-03	5.53
S100A16	S100 calcium binding protein A16	2.76E-03	2.68
KITLG	KIT ligand	2.90E-03	2.95
HGF	hepatocyte growth factor	2.96E-03	-3.39
CCL17	C-C motif chemokine ligand 17	3.93E-03	2.22
INHBA	inhibin beta A subunit	4.17E-03	4.89
PDGFD	platelet derived growth factor D	4.92E-03	-2.66
CXCL1	C-X-C motif chemokine ligand 1	5.21E-03	7.06
WNT5A	Wnt family member 5A	5.47E-03	4.95
WNT5B	Wnt family member 5B	5.81E-03	3.52
CSF1	colony stimulating factor 1	5.81E-03	2.29
CXCL2	C-X-C motif chemokine ligand 2	6.56E-03	5.98
CXCL3	C-X-C motif chemokine ligand 3	8.04E-03	4.32
IL16	interleukin 16	8.77E-03	-2.03

Table S5 Top 10 Proteins Binding to NRP-1

Gene names	Unique peptides IP	Sequence coverage [%]	Score	Sequence coverage IgG [%]	Sequence coverage IP [%]	Intensity IgG	Intensity IP
AP2B1	21	48.3	323.31	0	48.3	0	27116000000
AP2A1	30	42.9	323.31	0	42.9	0	20496000000
EPS15	25	34.4	323.31	0	34.4	0	3683000000
AAK1	12	34.8	223.64	0	34.8	0	2969000000
BMP2K	22	25	221.4	0	25	0	2896300000
AP2M1	15	34.6	203.38	0	34.6	0	12804000000
AP2A2	19	31.6	179.93	0	31.6	0	3488900000
GAPVD1	22	15.1	166.86	0	15.1	0	1938000000
TBC1D2B	15	18.7	128.84	0	18.7	0	1379700000
MTCL1	15	10	123.48	0	10	0	568670000

Table S6 Correlations Between NRP-1, GAPVD1 and Pathological Parameters in TNBC Patients

Variables	NRP-1 Staining		*P*	GAPVD1 Staining		*P*
	Low expression	High expression		Low expression	High expression	
Age						
< 51	11	17	> 0.99999	12	16	> 0.99999
≥51	8	14		9	13	
Location						
Left	4	8		3	9	
Right	7	15	> 0.99999	10	12	0.2919
Unknown	4	12		4	12	
Tumor Size						
T1-2	17	17	**0.0134***	18	16	**0.0321***
T3-4	2	14		3	13	
Lymph Node						
N0	18	21	**0.0352***	17	22	0.7412
N1	1	10		4	7	
Grade						
1-2	8	25		14	19	
3	5	5	0.1203	2	8	0.2757
Unknown	6	1		5	3	
Metastasis						
M0	19	31	> 0.99999	22	28	> 0.99999
M1	0	0		0	0	

Significant variables ($p < 0.05$) are bolded and *

Supplemental Table

Table S1 Differentially Expressed Genes in GEO dataset

Table S2 Secretory Factor Set Genes in GeneCard

Table S4 Proteins Binding to NRP-1
